# Fibroblast-Mediated Macrophage Recruitment Supports Acute Wound Healing

**DOI:** 10.1016/j.jid.2024.10.609

**Published:** 2024-11-22

**Authors:** Veronica M. Amuso, MaryEllen R. Haas, Paula O. Cooper, Ranojoy Chatterjee, Sana Hafiz, Shatha Salameh, Chiraag Gohel, Miguel F. Mazumder, Violet Josephson, Sarah S. Kleb, Khatereh Khorsandi, Anelia Horvath, Ali Rahnavard, Brett A. Shook

**Affiliations:** 1 The Department of Biochemistry & Molecular Medicine, School of Medicine & Health Sciences, The George Washington University, Washington, District of Columbia, USA;; 2 Computational Biology Institute, Department of Biostatistics and Bioinformatics, Milken Institute School of Public Health, The George Washington University, Washington, District of Columbia, USA;; 3 The Department of Dermatology, School of Medicine & Health Sciences, The George Washington University, Washington, District of Columbia, USA

**Keywords:** CCL2, Fibroblast, Macrophage, Single-nuclei RNA sequencing, Wound healing

## Abstract

Epithelial and immune cells have long been appreciated for their contribution to the early immune response after injury; however, much less is known about the role of mesenchymal cells. Using single-nuclei RNA sequencing, we defined changes in gene expression associated with inflammation 1 day after wounding in mouse skin. Compared with those in keratinocytes and myeloid cells, we detected enriched expression of proinflammatory genes in fibroblasts associated with deeper layers of the skin. In particular, SCA1+ fibroblasts were enriched for numerous chemokines, including CCL2, CCL7, and IL-33, compared with SCA1− fibroblasts. Genetic deletion of *Ccl2* in fibroblasts resulted in fewer wound-bed macrophages and monocytes during injury-induced inflammation, with reduced revascularization and re-epithelialization during the proliferation phase of healing. These findings highlight the important contribution of fibroblast-derived factors to injury-induced inflammation and the impact of immune cell dysregulation on subsequent tissue repair.

## INTRODUCTION

Skin wound healing requires intricate cell–cell communication to proceed effectively. The initial injury response generates a proinflammatory environment characterized by the release of inflammatory factors and the recruitment of myeloid cells (Gurtner et al, 2008; Ridiandries et al, 2018). Robust inflammation at this early stage of repair is critical because sufficient numbers of macrophages must be recruited to become essential mediators of tissue repair (Boniakowski et al, 2018; Goren et al, 2009; Lucas et al, 2010; Mirza et al, 2009; Sawaya et al, 2020; Shook et al, 2016; Willenborg et al, 2012; Wood et al, 2014). Insufficient or excessive inflammation and macrophage recruitment prevent the wound environment from shifting to a prohealing state that supports wound closure, precipitating the healing defects observed in conditions such as diabetes or aging (Audu et al, 2022; Eming et al, 2014; Gould et al, 2015; Joshi et al, 2020; Mirza and Koh, 2011; Mirza et al, 2014; Pang et al, 2021; Sawaya et al, 2020; Vu et al, 2022). This underscores the necessity for tight control of injury-induced inflammation and the need to define the cellular mediators of this stage of repair.

Tissue-resident cells help to regulate the proinflammatory environment after injury. Keratinocytes (KCs) are a well-established source of inflammatory cytokines and immune cell chemoattractants (Lebre et al, 2007; Villarreal-Ponce et al, 2020). Dermal adipocytes produce adipokines and lipids, which can modulate wound macrophage numbers and impact wound repair (Shook et al, 2020; Zhang et al, 2019). In addition, the adoption of a proinflammatory gene signature was recently established as a critical component of fibroblast activity during wound healing (Almet et al, 2024; Correa-Gallegos et al, 2023). These findings corroborate that injury-induced inflammation is a concerted effort from the plethora of cell types in the skin and highlight that further research is necessary to define specific roles for nonimmune cells during this stage of healing.

To contextualize the changes in gene expression that occur in the different skin cell types after injury, we performed unbiased single-nuclei RNA-sequencing (snRNA-seq) analysis of murine skin wounds 1 day after wounding. Although KCs and macrophages upregulated genes associated with immune cell chemotaxis to wounds, we found the greatest increase in proinflammatory gene expression within SCA1+ fibroblasts, which are more prevalent in deeper layers of the skin (Driskell et al, 2013; Lichtenberger et al, 2016; Sun et al, 2023). SCA1+ fibroblasts were enriched for expression of inflammatory chemokines, such as CCL2 and CCL7, and genetic ablation of *Ccl2* from fibroblasts resulted in dramatic decreases in wound macrophage and monocyte numbers during inflammation with impaired tissue healing. These findings establish fibroblasts as key mediators of injury-induced inflammation that produce essential factors for immune cell recruitment and progression through wound healing.

## RESULTS

### Stromal cells acquire a proinflammatory transcriptional state after injury

**Figure 1.**
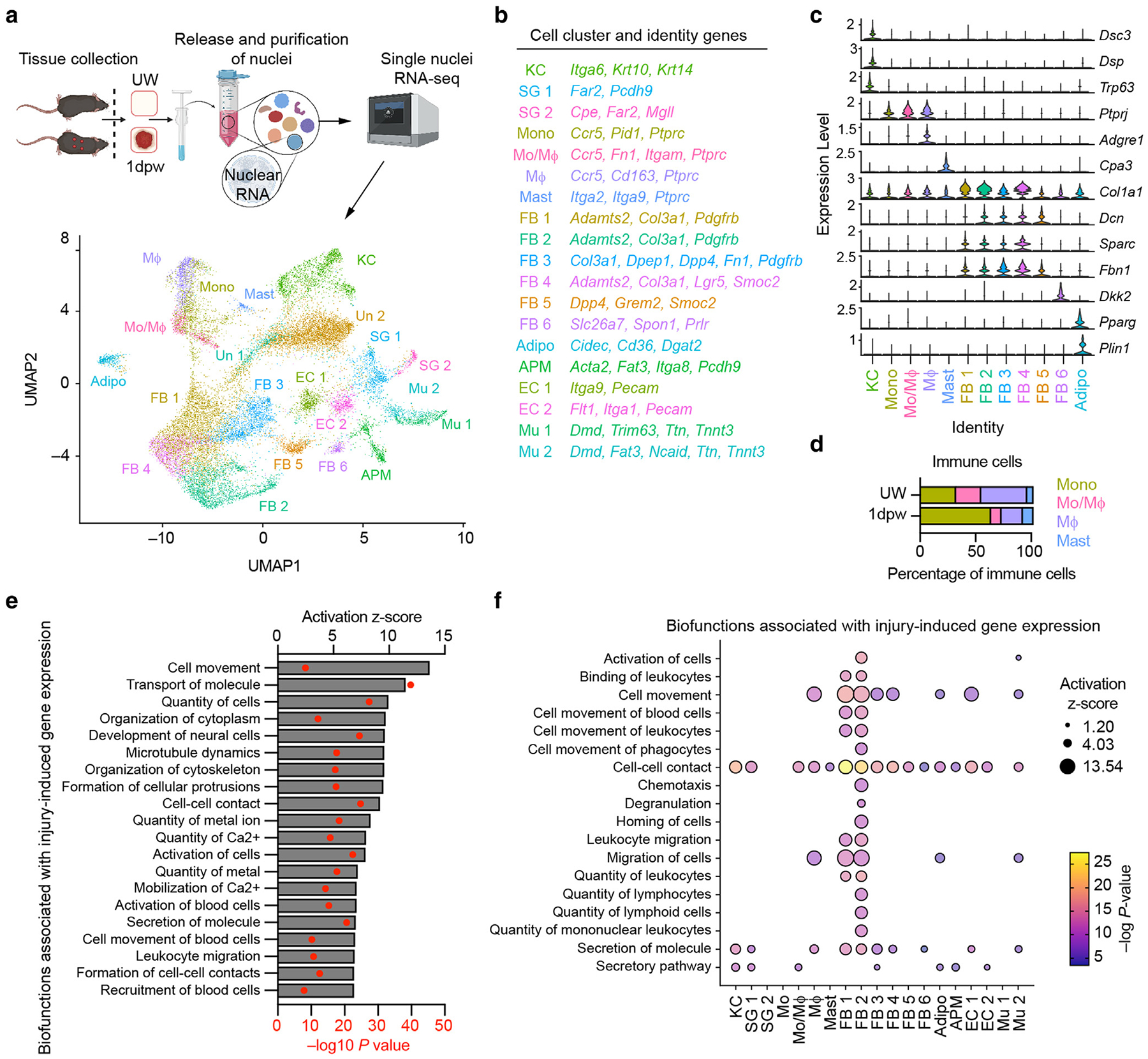
Epithelial, stromal, and immune cells adopt a proinflammatory gene expression profile after skin injury. (**a**) Schematic of nuclei harvested from uninjured and wounded skin for snRNA-seq (n = 2 mice per group) and UMAP analysis of snRNA-seq data. (**b**) Identification of genes for cell clusters. (**c**) Violin plot for genes associated with specific cellular identities. (**d**) Frequency of immune cell clusters in cells from UW skin and 1 dpw. (**e**) Activated biofunctions associated with genes upregulated 1 dpw. (**f**) Bubble matrix of biofunctions associated with upregulated gene expression in each cell cluster 1 dpw. dpw, day after wounding; snRNA-seq, single-nuclei RNA sequencing; UMAP, Uniform Manifold Approximation and Projection; UW, unwounded.

Diverse intercellular crosstalk pathways are transiently activated to produce proinflammatory and then prohealing conditions at the injury site (Brazil et al, 2019; Eming et al, 2014; Gurtner et al, 2008; Shook et al, 2020, 2018; Vu et al, 2022). Single-cell RNA sequencing is a powerful identifier of cellular heterogeneity and predictive cellular function; however, cells that are fragile or tightly embedded in the extracellular matrix are frequently absent from generated datasets (Habib et al, 2017). To circumvent this limitation, we performed snRNA-seq on nuclei isolated from unwounded skin and the wound periphery from full-thickness excisional wounds (Figure 1a). Because robust populations of neutrophils, monocytes, and macrophages are present in wounds 1.5 days after wounding (Shook et al, 2020), we isolated nuclei from tissues 24 hours (1 day after wounding) to interrogate nuclear mRNA that could contribute to the recruitment or polarization of cells present at 1.5 days after wounding.

We identified 21 cell clusters from the snRNA-seq data (Figure 1a and Supplementary Figure S1a and b). Using canonical marker genes, we classified clusters as specific epithelial, immune, or stromal cell types (Figure 1a–c and Supplementary Figure S1c). Three epithelial cell clusters were identified as KCs (*Dsc3*, *Dsp*, and *Trp63*) (Puzzi et al, 2018; Qu et al, 2018; Spindler et al, 2009) or sebaceous gland cells (SG1 and SG2) (*Far2*) (Konger et al, 2021; Rittié et al, 2016; Sundberg et al, 2018). Four immune cell populations (enriched for *Ptprc*) were classified as mast cells (*Cpa3*) (Atiakshin et al, 2022; Siddhuraj et al, 2021) and 3 clusters of monocytes/macrophages (*Ptprj* and *Ccr5*) (Castanheira et al, 2019; Dave et al, 2009; Oppermann, 2004; Osborne et al, 1998). Within the monocyte/macrophage groups, we identified mature macrophages (Mɸ) (high *Adgre1* and *Cd163)* (Jo et al, 2021; Pang et al, 2022), monocytes (*Pid1)* (Lorenz et al, 2019), and a population that appeared to be immature monocyte-derived macrophages (Mo/Mɸ) (intermediate *Adgre1*). Interestingly, monocytes comprised over 50% of the immune cell population in the 1-day-post-wounding samples, characteristic of monocyte influx to injury during inflammation (Figure 1d) (Crane et al, 2014). Notably, we did not detect a neutrophil cluster, although neutrophils are quickly recruited to wounds (de Oliveira et al, 2016; Shook et al, 2020). Their absence likely resulted from their generally lower RNA content and direct exposure to endogenous nucleases during tissue processing (Maraux et al, 2020; Wigerblad et al, 2022). Within the 14 stromal cell clusters, we identified endothelial cells (EC1 and EC2) (*Pecam1*) (Tombor et al, 2021), skeletal muscle clusters (Mu1 and Mu2) (*Dmd*) (Hoffman et al, 1987), arrector pili muscle cells (*Acta2*, *Itga8*) (Ahlers et al, 2021; Fujiwara et al, 2011), adipocytes (*Cidec*, *Plin1*, and *Pparg*) (Kim et al, 2008; Rosen et al, 1999; Shook et al, 2020), and 6 fibroblast populations (FB1–FB6) (*Col3a1*, *Dcn*, *Sparc*, and *Fbn1*) (He et al, 2023; Joost et al, 2020; Philippeos et al, 2018; Shook et al, 2020). Two clusters were not clearly enriched for markers associated with a particular cell type and were classified as unknown (Un1 and Un2).

Fibroblasts from different layers of the skin exhibit distinct gene expression profiles and perform unique functions (Almet et al, 2024; Correa-Gallegos et al, 2023; Driskell et al, 2013; Frech et al, 2022; Guerrero-Juarez et al, 2019; Janson et al, 2012; Jiang et al, 2020; Korosec et al, 2019; Philippeos et al, 2018). We endeavored to distinguish the 6 fibroblast subsets by interrogating their expression of genes associated with fibroblasts in different skin layers. One cluster (FB6) was enriched for the papillary dermis and dermal papilla markers *Dkk2*, *Lef1*, *Spon1*, and *Prlr* (Liu et al, 2022; Phan et al, 2020) (Figure 1b and Supplementary Figure S1c). Whereas fibroblast clusters 1–5 shared expression of the dermal fibroblast markers *Dcn* and *Sparc* (Joost et al, 2020), FB5 had a greater enrichment for *Grem2* and *Smoc2*, suggesting that they are reticular dermal fibroblasts (Phan et al, 2020) (Figure 1b and Supplementary Figure S1c). Although a more granular prediction of cluster localization in the skin based on markers associated with the reticular dermis, hypodermis, and fascia was not possible owing to the low nuclear RNA counts, the expression pattern of these genes suggests that FB1–4 reside in the dermis and deeper layers of the skin such as the hypodermis/fascia.

Significant injury-induced changes in gene expression across all cells were defined using Tweedieverse, a differential expression tool that calculates an effect size by considering the distribution and dispersion of gene expression, thereby improving the modeling of overdispersion and zero inflation in single-cell RNA-sequencing analyses (Mallick et al, 2022). The potential biofunctions regulated by genes with a significant Tweedieverse effect size 1 day after wounding were determined using Ingenuity Pathway Analysis (Krämer et al, 2014). These included cell movement, leukocyte migration, secretion of molecules, and recruitment of blood cells (Figure 1e), reflecting cytokine and chemokine production that recruits myeloid cells to the wound. Indeed, predicted upstream signaling inducing these biofunctions included a variety of cytokines and their receptors (Supplementary Figure S1d), and numerous proinflammatory cytokines had a significant Tweedieverse effect size 1 day after wounding (Supplementary Figure S1e).

We further investigated the biofunctions associated with the 1-day-postwounding gene expression signature for each cell cluster (Figure 1f and Supplementary Figure S2a and b). Predicted biofunctions related to cell morphology, development, organization (Supplementary Figure S2a), cell signaling, molecular transport, metabolism, and cell maintenance (Supplementary Figure S2b) were shared among many populations; however, biofunctions related to immune cell recruitment were more restricted to specific cell types. Mo/Mɸ, FB1, and FB2 clusters showed the greatest predicted activation of biofunctions associated with immune cell recruitment, such as chemotaxis and quantity of leukocytes (Figure 1f). To explore active cellular communication networks in our dataset, we performed CellChat analysis (Jin et al, 2024, 2021). Although the top signaling pathways predicted to be active were NRG, SEMA3, SLIT, fibroblast GF, and BMP signaling (Supplementary Figure S2c), most of these pathways had greater predicted activation in nonimmune cells. In line with previous reports (Boniakowski et al, 2018; Willenborg et al, 2012; Wood et al, 2014), CCL signaling was predominantly active in monocyte/macrophage clusters (Supplementary Figure S2d). Surprisingly, the predicted ligande–receptor interactions did not include CCL2–CCR2 owing to the low detection of *Ccr2* in monocyte/macrophage clusters (Supplementary Figure S2e and f). Although we detected more robust expression of ligands (Supplementary Figure S2g), the lack of robust detection of receptor expression is a limitation of our dataset that prevents the utilization of sophisticated computational tools, such as CellChat, to predict cellular communication.

**Figure 2.**
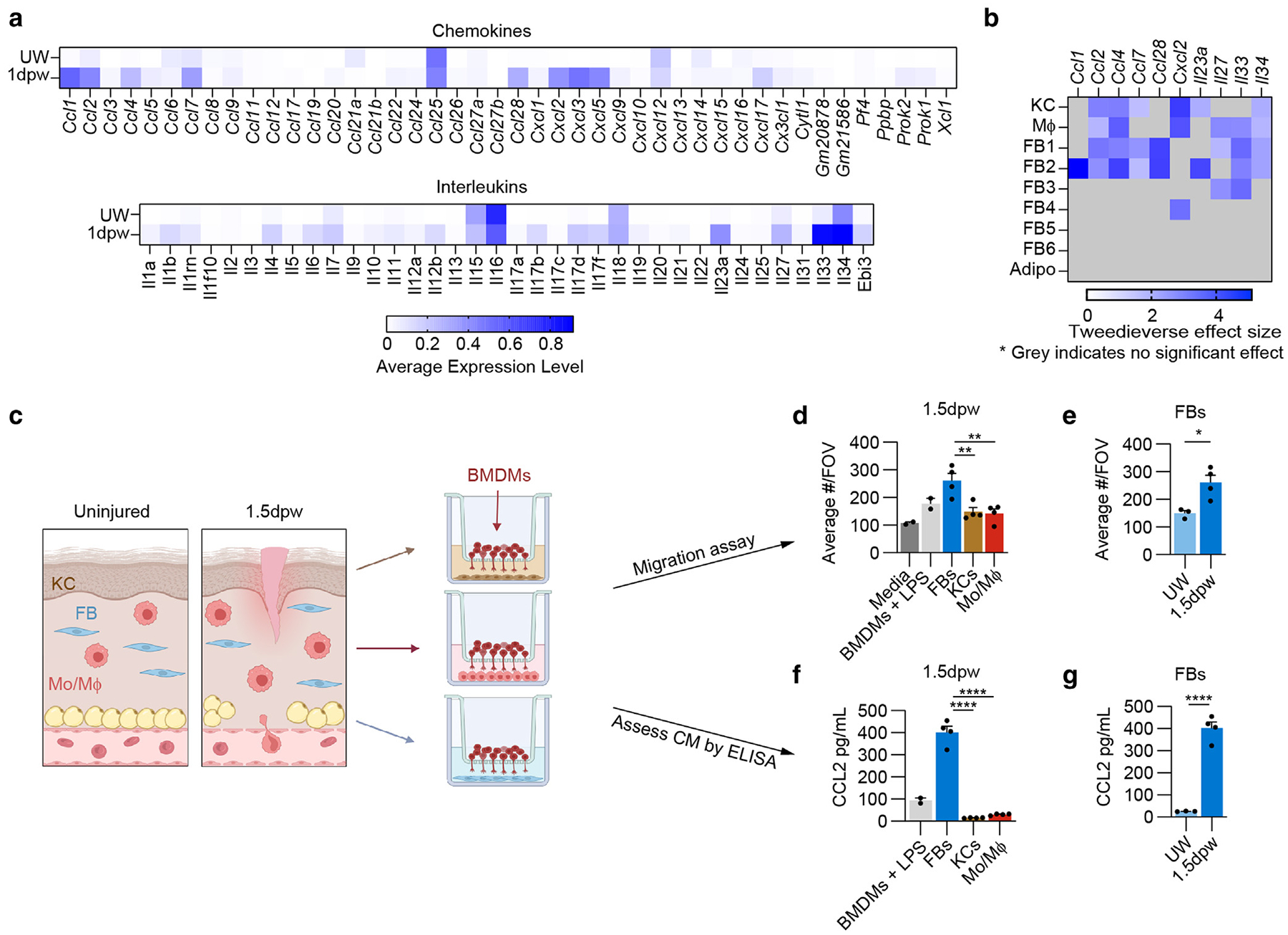
Multiple FB subsets acquire a proinflammatory cellular state 24 hours after injury. (**a**) Average expression of cytokines and ILs in the UW and wounded (1 dpw) conditions. (**b**) Cytokines with a significantly increased Tweedieverse effect size in multiple cell types 1 dpw. (**c**) Schematic of BMDM migration assay with primary FBs (CD45−, CD31−, EPCAM−), KCs (CD45−, EPCAM+), and monocytes/macrophages (denoted as Mo/Mɸ) (CD45+, CD11b+, Ly6G−). (**d, e**) Quantification of the number of BMDMs per 20× FOV that migrated to the lower portion of the transwell. (**f, g**) Quantification of CCL2 in the CM at the endpoint of the BMDM migration assay. n = 3 mice per group for conditions with primary cells and 2 technical replicates for the media and BMDMs + LPS control conditions. Error bars indicate mean ± SEM. *P < .05, **P < .01, and ****P < .0001. BMDM, bone marrow–derived macrophage; CM, conditioned media; dpw, day after wounding; FB, fibroblast; FOV, field of view; KC, keratinocyte; LPS, lipopolysaccharide; UW, unwounded.

Because the low sequencing depth associated with snRNA-seq of neutrophil-rich samples makes it challenging to perform predictive cell communication analysis, we explored our dataset to identify genes that may contribute to the inflammatory environment after injury and the specific processes identified in our biofunction analysis. To do this, we examined the average expression of chemokines and ILs in the unwounded and 1-day-postwounding samples (Figure 2a). The 1-day-postwounding samples generally displayed a higher average expression of these factors than the unwounded condition. To focus our investigation on the factors most likely to influence inflammation and immune cell modulation, we excluded from further analysis any genes with an average expression level <0.2 in both conditions. From here, we explored genes with significant increases in gene expression 1 day after wounding in individual cell populations using Tweedieverse. Because KCs, macrophages, fibroblasts, and adipocytes may all contribute to injury-induced inflammation (Correa-Gallegos et al, 2023; Daley et al, 2010; He et al, 2023; Lebre et al, 2007; Pang et al, 2022; Shook et al, 2020; Villarreal-Ponce et al, 2020; Zhang et al, 2019), we focused on these cells to investigate changes in gene expression capable of influencing the immune response. Using KCs and macrophages as a baseline to explore injury-induced proinflammatory gene expression, we observed multiple cytokines significantly upregulated by fibroblasts after injury (Figure 2b). Most notably, *Ccl2*, *Ccl4*, *Ccl7*, and *Il33* had a significant effect size in multiple fibroblast clusters, with FB1 and FB2 having the greatest number of cytokines upregulated after injury. Similarly, fibroblasts upregulated many GFs, extracellular matrix molecules, and extracellular matrix modifiers after injury, with some shared by epithelial and immune cells and some unique to fibroblasts (Supplementary Figure S3a–c). Interestingly, we did not detect many significant increases in cytokines or GFs in our adipocyte population; however, low nuclear transcript counts could result in false negatives within the snRNA-seq analysis. Our gene expression analysis highlights the redundant nature of proinflammatory gene expression profiles among cellular populations and corroborates fibroblasts as a putative source of proinflammatory cues during early wound healing (Almet et al, 2024; Correa-Gallegos et al, 2023; Sinha et al, 2022).

Given that many of the differentially expressed proinflammatory genes were chemoattractants, we evaluated the ability of KCs, monocyte/macrophages, and fibroblasts to induce bone marrow–derived macrophage (BMDM) migration in vitro (Figure 2c) and used lipopolysaccharide-stimulated BMDMs as a positive control for cells that generate chemoattractants. KCs, monocytes/macrophages, and fibroblasts were isolated from 1.5 days after wounding to allow time for nuclear RNA expression detected in the 1-day-postwounding snRNA-seq data to be converted into functional protein. Interestingly, fibroblasts induced the greatest amount of BMDM migration (Figure 2d), and fibroblast-mediated induction of BMDM migration was injury dependent (Figure 2e). Assessment of the cell conditioned media by ELISA confirmed that 1.5-day-postwounding fibroblasts release significant amounts of the chemokine CCL2 compared with 1.5-day-postwounding KCs and monocytes/macrophages (Figure 2f) and fibroblasts isolated from uninjured skin (Figure 2g). These data suggest that fibroblasts can impact essential inflammatory processes, such as immune cell recruitment, thereby supporting their role as inflammatory mediators during healing.

### Distinct fibroblast populations have enriched expression of macrophage chemoattractants after injury

**Figure 3.**
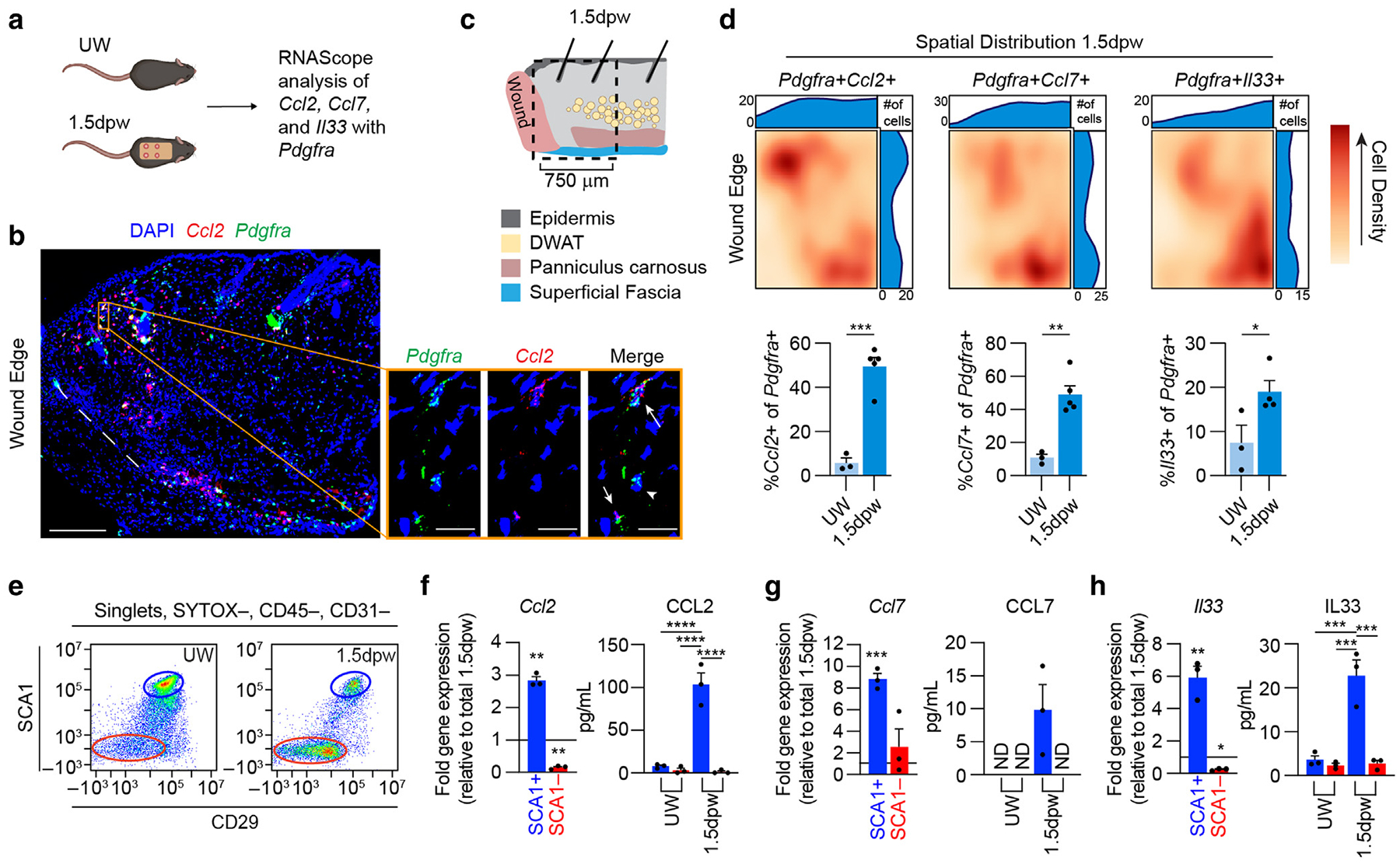
SCA1+ fibroblasts are enriched for inflammatory gene expression. (**a**) Schematic of RNAscope assessment of fibroblasts (*Pdgfra+*) expressing *Ccl2*, *Ccl7*, or *Il33* in UW skin and at the wound periphery 1.5 dpw. (**b**) Representative composite image of the wound periphery 1.5 dpw with *Ccl2* (red) and *Pdgfra* (green) signal. The white dashed line delineates the wound edge. High magnification images (right) correspond to the orange boxed area in the left image, with double-positive (*Pdgfra*+*Ccl2*+) and single-positive (*Pdgfra*+) cells highlighted by arrows and arrowheads, respectively. Bars = 200 μm (left image) and 20 μm (right images). (**c**) Schematic of wound periphery area analyzed for RNAScope. (**d**) Top: spatial analysis of *Ccl2+*,*Ccl7+*, or *Il33+* fibroblasts at the wound periphery. Heatmaps show the distribution of double-positive cells in the area corresponding to the boxed region of the diagram in **c**. Bottom: percentage of *Pdgfra*+ cells expressing *Ccl2*, *Ccl7*, or *Il33* in the regions analyzed 1.5 dpw and in 750-μm-wide sections of UW tissue. n = 3 tissue sections (UW) or ≥ 4 wound edges (1.5 dpw) from 3 different mice per group. (**e**) Flow cytometry plots of SCA1 and CD29 levels in live, CD45−, CD31−, EPCAM cells. (**f**–**h**) RT-qPCR relative fold change gene expression and ELISA quantification of supernatant from sorted CD45–, CD31–, EPCAM–, SCA1+ and CD45−, CD31−, EPCAM−, SCA1− fibroblasts for (**f**) *Ccl2*/CCL2, (**g**) *Ccl7*/CCL7, and (**h**) *Il33*/IL33. Data are normalized to the expression levels in total tissue 1.5 dpw. n = 3 mice per group. Error bars indicate mean ± SEM. *P < .05, **P < .01, ***P < .001, and ****P < .0001. dpw, day after wounding; UW, unwounded.

Our 1-day-postwounding snRNA-seq data indicate that fibroblasts FB1 and FB2 generate proinflammatory signaling molecules capable of influencing macrophage infiltration during the early stages of wound healing (Figures 1f and 2b). To explore the spatial location of inflammatory fibroblasts in the skin, we used RNAscope to assess genes upregulated by these fibroblasts. We quantified *Ccl2*, *Ccl7*, and *Il33* in *Pdgfra*+ fibroblasts at the wound periphery 1.5 days after wounding to capture greater quantities of mature mRNA (Figure 3a and b and Supplementary Figure S4a and b). Fibroblasts significantly increased expression of all 3 genes 1.5 days after wounding, and *Ccl2*, *Ccl7*, and *Il33+* fibroblasts were biased toward the superficial dermis near the wound edge and deeper in the skin, around the panniculus carnosus and superficial fascia (Figure 3c and d). These results support the previous finding that fibroblasts in the fascia adopt a proinflammatory profile after injury (Correa-Gallegos et al, 2023). Fibroblasts in the deep dermis and underlying fascia are enriched for expression of SCA1 (*Ly6a*) (Driskell et al, 2013; Lichtenberger et al, 2016; Sun et al, 2023), and the 5-day-postwounding transcriptomic profile of SCA1+ fibroblasts is enriched for secreted factors that can modulate macrophage migration and function (Shook et al, 2018). Several of these cytokines were identified in the 1-day-post-wounding fibroblast clusters 1 and 2, including *Ccl2*, *Ccl7*, and *Il33* (Figure 2b), supporting the idea that functional diversity may exist among skin fibroblasts during inflammation.

To further validate that injury-induced proinflammatory gene expression is not uniform across all fibroblasts, we isolated lineage-negative stromal cells (CD45−, CD31−) on the basis of SCA1 and CD29 surface levels 1.5 days after wounding (Figure 3e and Supplementary Figure S4c) (Haas et al, 2021; Shook et al, 2018). SCA1+ fibroblasts were significantly enriched for expression of *Ccl2*, *Ccl7*, *Cxcl1*, *Cxcl12*, *Il6*, and *Il33* 1.5 days after wounding compared with the expression level in the entire wound bed (Figure 3f–h and Supplementary Figure S4d). The expression level of these genes in the SCA1− population was not significantly different or was significantly lower (Figure 3f–h and Supplementary Figure S4d). Secreted protein analysis corroborated the gene expression data, indicating that SCA1+ fibroblasts generate significantly more CCL2, CCL7, and IL-33 than SCA1− cells as well as significantly more than SCA1+ fibroblasts from uninjured skin (Figure 3f–h). Overall, these results support that stromal cells, particularly SCA1+ fibroblasts, are a key local source of inflammatory factors during early injury-induced inflammation.

### Fibroblast-derived CCL2 is required for injury-induced macrophage recruitment

Signaling through CCR2 is essential for monocyte and macrophage trafficking to the wound (Boniakowski et al, 2018; Willenborg et al, 2012; Wood et al, 2014). The upregulation of fibroblast *Ccl2* in our snRNA-seq data and the enriched expression and secretion of *Ccl2*/CCL2 in SCA1+ fibroblasts implicate CCL2 as a key mediator of fibroblast–macrophage communication during injury-induced inflammation. To assess how fibroblast-derived CCL2 impacts immune cell numbers in the wound, we crossed *Pdgfra*CreER mice (Chung et al, 2018) with *Ccl2* floxed mice (Shi et al, 2011) to genetically target *Ccl2* in fibroblasts. Administration of tamoxifen to *Pdgfra*CreER+ mice selectively targets fibroblast subsets in the skin (Supplementary Figure S5a and b), allowing for the temporally regulated deletion of *Ccl2* in fibroblasts. SCA1+ fibroblasts isolated from *Pdgfra*CreER+; *Ccl2*^fl/fl^ (fibroblast conditional knockout [FBcKO]) mice 16 hours after wounding and 1.5 days after wounding displayed reduced *Ccl2* expression compared with *Pdgfra*CreER-; *Ccl2*^fl/fl^ (control) mice (Supplementary Figure S5c and d). Although SCA1− fibroblasts express minimal *Ccl2* relative to the SCA1+ fibroblasts (Figure 3f), we detected a reduction in *Ccl2* expression in SCA1− fibroblasts isolated from wound beds of FBcKO mice 1.5 days after wounding (Supplementary Figure S5d). This suggests that SCA1− fibroblasts may increase expression of *Ccl2* at a later time point after injury than SCA1+ fibroblasts or SCA1 expression in fibroblasts may change on the basis of inflammation, similar to what has previously been shown during wound healing (Shook et al, 2018) and different stages of the hair cycle (Lichtenberger et al, 2016).

**Figure 4.**
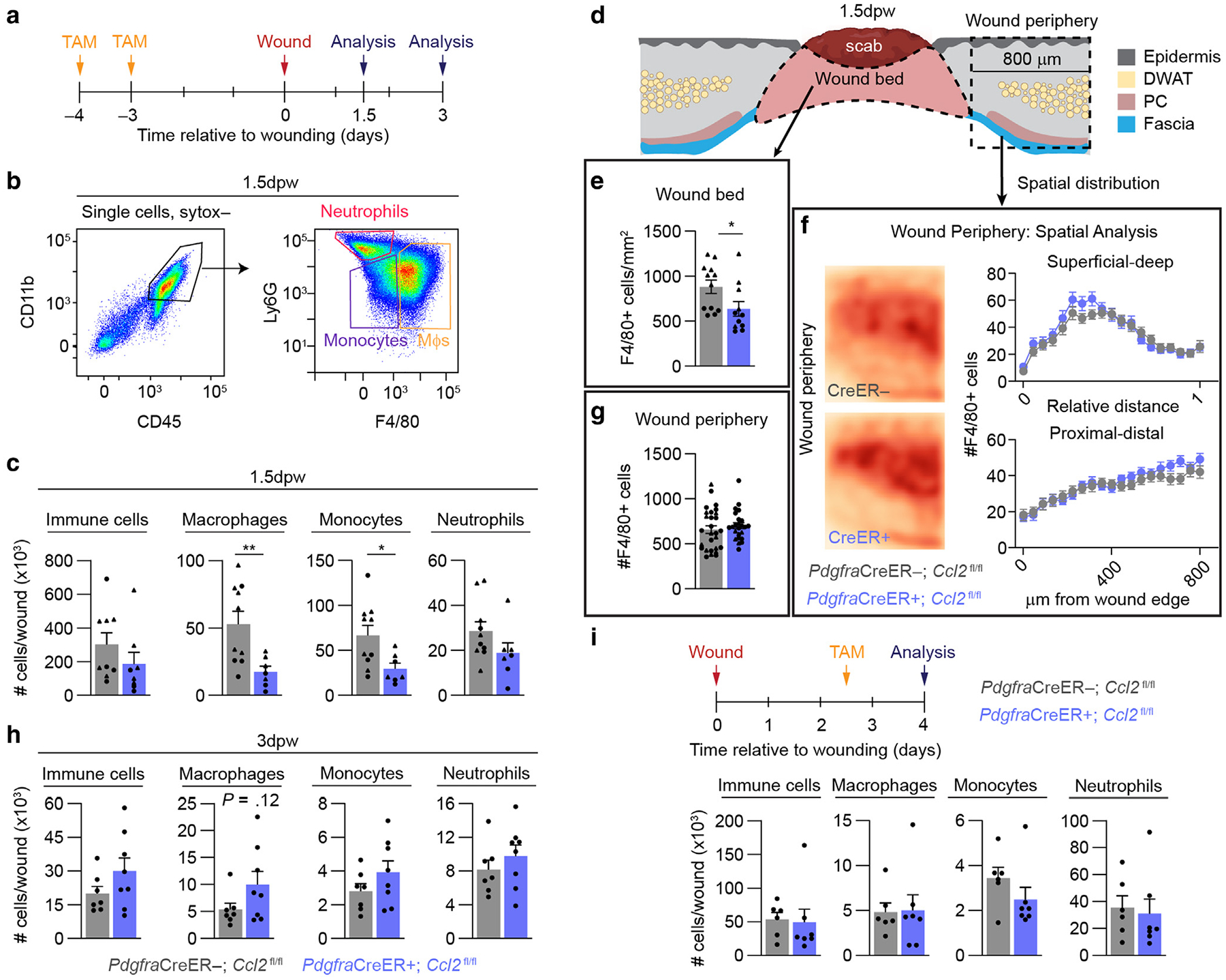
Fibroblast-derived CCL2 supports macrophage numbers during the inflammation phase of wound healing. (**a**) Paradigm to induce *Ccl2* deletion in *Pdgfra*-expressing cells and analysis time points in *Pdgfra*CreER+; *Ccl2*^fl/fl^ mice (FBcKO) and *Pdgfra*CreER−; *Ccl2*^fl/fl^ (control) mice. (**b**) Gating strategy for immune cell populations in wound beds. (**c**) Flow cytometry quantification of immune cell subsets in FBcKO and control mice. n = 7 mice per group. (**d**) Schematic of spatial tissue analysis of macrophages (F4/80+) in FBcKO and control mice. (**e**) Quantification of F4/80+ cells in the wound centers. n = 7 wounds from ≥7 different mice per group. (**f, g**) Quantification of F4/80+ cells at the wound periphery. (**f**) Heatmaps and graphed distribution of F4/80+ cells along the superficial–deep and proximal–distal axes. (**g**) Total numbers of F4/80+ cells in the wound periphery. For **f** and **g**, n = 21 wound edges from 7 mice per group. (**h**) Flow cytometry quantification of immune cell subsets at 3 dpw. n = 7 mice per group. (**i**) Timeline and immunophenotyping quantification for tamoxifen administration to FBcKO and control mice at the end of the inflammatory phase. n = 6 mice per group. Triangles and circles delineate female and male mice, respectively. Error bars indicate mean ± SEM. *P < .05 and **P < .01. dpw, day after wounding; DWAT, dermal white adipose tissue; FBcKO, fibroblast conditional knockout; PC, panniculus carnosus.

We evaluated the effect of fibroblast-derived CCL2 on immune cell numbers during the inflammatory phase at 1.5 days after wounding and 3 days after wounding (Figure 4a and b). At 1.5 days after wounding, FBcKO mice displayed greater than a 50% reduction in wound macrophage and monocyte numbers than control mice (Figure 4c), suggesting a profound defect in myeloid cell trafficking to the wound. Neutrophils and total CD45+ immune cells were also slightly reduced. Immunostaining for the macrophage marker F4/80 in 1.5-day-postwounding tissue sections revealed that the reduction in F4/80+ macrophages was limited to the wound bed in FBcKO mice (Figure 4d–g and Supplementary Figure S5e and f). Supporting a direct effect of CCL2-mediated chemotaxis, there were significantly fewer CCR2+ monocytes/macrophages in wound beds of FBcKO mice (Supplementary Figure S5g–i). Interestingly, SCA1+ fibroblasts from FBcKO mouse wounds possessed diminished yet not significant in vitro macrophage chemotactic potential compared with fibroblasts from control mice (Supplementary Figure S5j). Furthermore, 3 days after wounding, the trends in immune cell numbers had switched because FBcKO mice tended to have higher numbers of immune cells, macrophages, and monocytes per wound than control mice, although the data did not reach statistical significance (Figure 4h). To explore this apparent recovery in the FBcKO mice, we evaluated gene expression in SCA1+ and SCA1− fibroblasts for the chemokines *Ccl2* and *Ccl7*, which also signals through CCR2 (Tsou et al, 2007; Willenborg et al, 2012), 16 hours after wounding and 1.5 days after wounding (Supplementary Figure S5c and d). Sixteen hours after wounding, *Ccl2* expression was lower in the total wound and SCA1+ fibroblasts from FBcKO mice than in the controls, and *Ccl7* expression was not significantly different between the groups. However, 1.5 days after wounding, the difference in *Ccl2* expression in the total wound was no longer significant, and *Ccl7* expression was higher on average in the SCA1+ fibroblasts from FBcKO mice. This compensatory increase in *Ccl7* was not observed in the SCA1− fibroblasts. We further investigated *Ccl2* and *Ccl7* gene expression in KCs, monocytes, and macrophages 1.5 days after wounding (Supplementary Figure S5d). No difference in either of these chemokines was observed between the control and FBcKO mice in any cell group. Together, these results suggest that loss of CCL2 production from the SCA1+ fibroblasts impacts wound myeloid cell numbers in the earliest stages of inflammation, but compensatory increases in chemokine expression from these cells eventually allow for robust recruitment of myeloid cells.

We also explored whether fibroblast-derived CCL2 supports the continued recruitment of monocytes and macrophages to the wounds after peak inflammation. Knockout of *Ccl2* in fibroblasts 2.5 days after wounding did not alter the number of immune cells present in wounds 4 days after wounding (Figure 4i). This further emphasizes the role of SCA1+ fibroblast-derived CCL2 early after injury and suggests that fibroblasts play an essential role in the rapid, early recruitment of innate immune cells to the wound.

### Skin wound healing is impaired in fibroblast *Ccl2*-knockout mice

**Figure 5.**
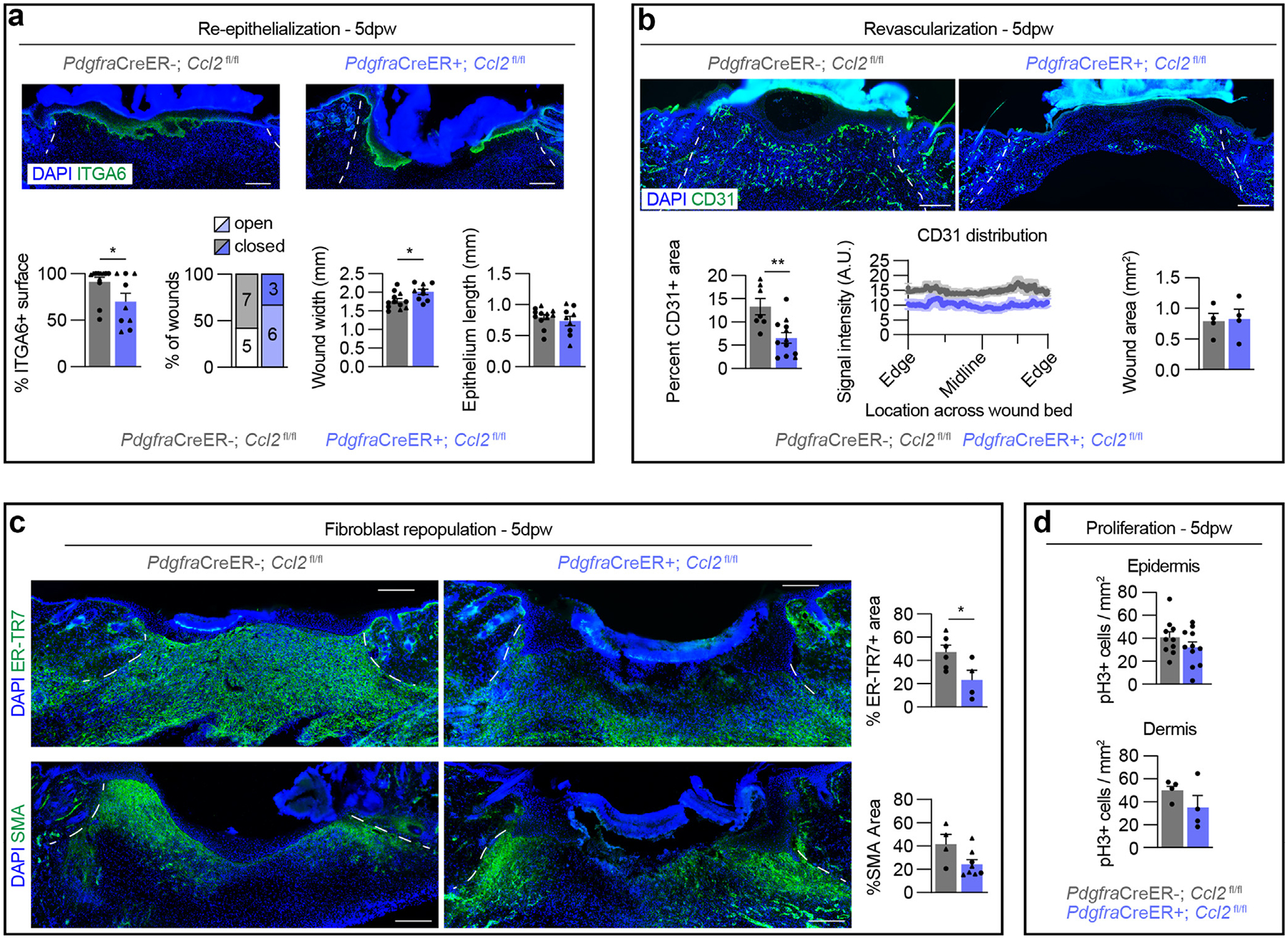
Epidermal and dermal repair are delayed in fibroblast *Ccl2*-knockout mice. (**a**) Images of wound beds of FBcKO and control mice 5 dpw immunostained for ITGA6 and DAPI. Graphs show the percentage of ITGA6+ surface, the frequency of open or closed wounds, wound width, and wound epithelium length. n = 9 wounds from 6 mice per group. (**b**) Images of wound beds of FBcKO and control mice 5 dpw immunostained for CD31 and DAPI. Graphs show the CD31+ area (left), the average distribution of CD31 signal intensity from the wound edge to the center (midline), and the total wound bed area (right). n = 4 wounds from 3 mice per group. (**c**) Images and quantification from wound beds of FBcKO and control mice 5 dpw immunostained for ERTR7 (top) and SMA (bottom) DAPI. n = 4 wounds from 3 mice per group. (**d**) Quantification of pH3+ cells in the epidermal (left) and dermal (right) compartments of wound beds 5 dpw in FBcKO and control mice. n = 4 wounds from 3 mice per group. Bars = 250 μm, and white lines delineate wound edges. Data points indicate individual wounds. Triangles and circles delineate female and male mice, respectively. Error bars indicate mean ± SEM. *P < .05 and **P < .01. A.U. denotes arbitrary units of fluorescence. dpw, day after wounding; FBcKO, fibroblast conditional knockout; pH3, phospho-histone H3; SMA, smooth muscle actin.

Directly reducing macrophage numbers during the inflammation phase delays revascularization, re-epithelialization, and granulation tissue formation (Goren et al, 2009; Lucas et al, 2010; Mirza et al, 2009; Shook et al, 2016; Willenborg et al, 2012). To determine whether the reduction in wound bed macrophages in FBcKO mice affects downstream wound healing, we examined multiple reparative processes in wounds 5 days after wounding. Immunostaining tissue sections from the center of wound beds with ITGA6 (Yan et al, 2022) revealed that re-epithelialization of wound beds was reduced in FBcKO mice, with fewer wounds becoming fully re-epithelialized or closed 5 days after wounding compared with controls (Figure 5a). Although the length of the migrated epithelium on each side of the wound was the same between groups, the wound width was slightly higher in the FBcKO group, suggesting that these mice may have altered wound contraction (Chen et al, 2015; Lucas et al, 2010; Parfejevs et al, 2018).

We investigated whether FBcKO mice displayed delayed healing in the dermal compartment. Compared with that in the control mice, revascularization was reduced throughout the wound bed in the FBcKO mice by 50%, although the average wound bed area was the same (Figure 5b). We also evaluated fibroblast repopulation of the wound bed with ERTR7 and myofibroblast presence with smooth muscle actin (Cooper et al, 2024; Haas et al, 2021). Wound beds from FBcKO mice displayed significantly reduced levels of ERTR7; yet, the smooth muscle actin–positive wound bed area was similar (Figure 5c). Notably, we did not observe a difference in the number of phospho-histone H3 (pH3)+ mitotic cells in the epidermis or dermis 5 days after wounding (Figure 5d). These results suggest that although induction of smooth muscle actin expression is unchanged in FBcKO mice, myofibroblast tension or tension from the panniculus carnosus is likely altered, leading to changes in wound contraction in FBcKO mice.

Because macrophage numbers eventually recovered in the FBcKO model, we evaluated wound-healing parameters 7 days after wounding to determine whether these defects also recovered. Although revascularization remained decreased in the FBcKO mice, we no longer observed differences in the other epithelial or fibroblast repopulation (Supplementary Figure S6a–d). Overall, the significant decrease in wound myeloid cell numbers during inflammation and subsequent healing delay observed in the fibroblast *Ccl2*-knockout mice highlight that fibroblast proinflammatory signaling is essential for timely progression through the healing process.

## DISCUSSION

**Figure 6.**
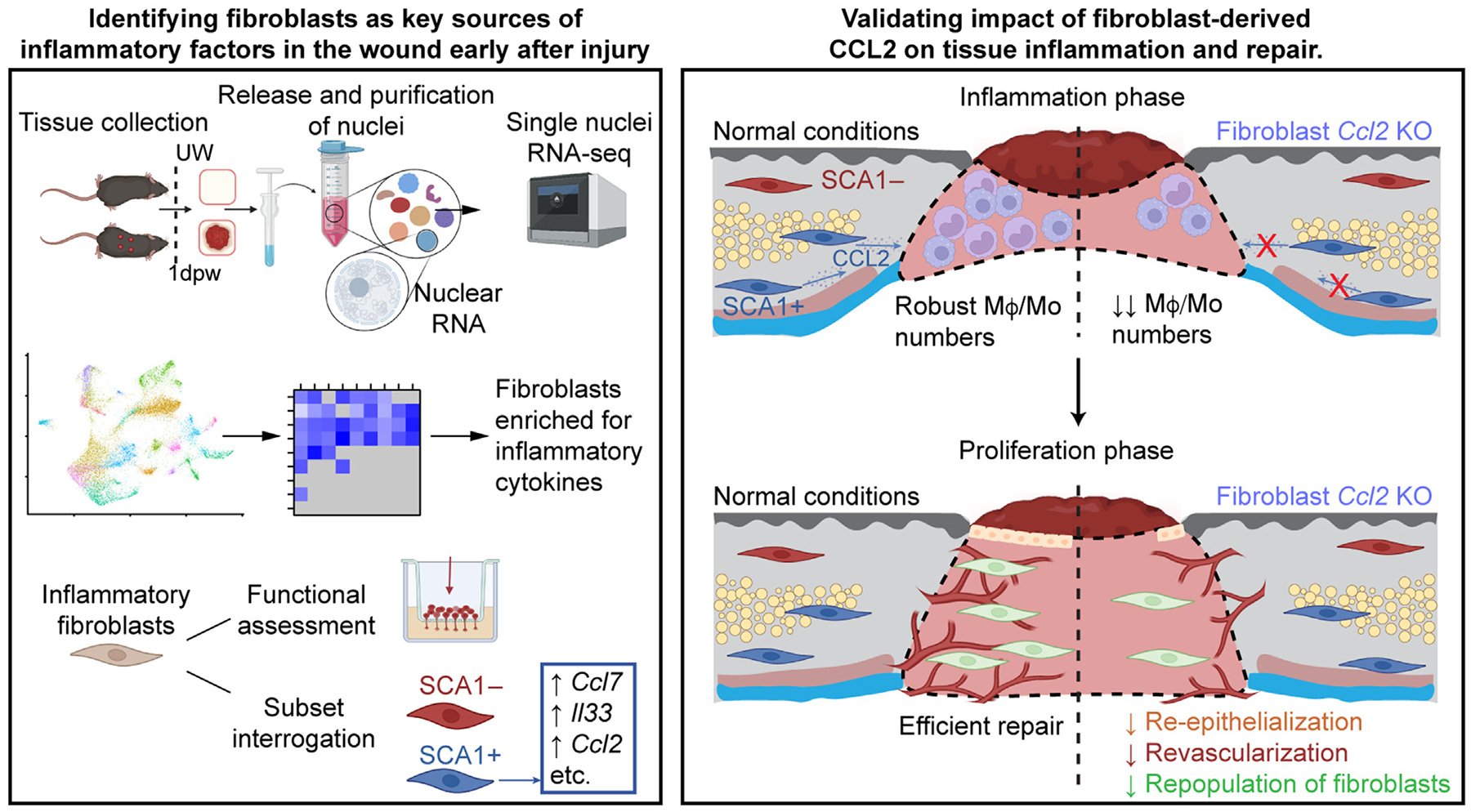
SCA1+ fibroblasts produce inflammatory factors during the early injury response that are critical for immune cell recruitment and subsequent tissue repair. KO, knockout; RNA-seq, RNA sequencing.

Mesenchymal cells are increasingly recognized as key coordinators of immune cell activity at homeostasis and after injury (Krausgruber et al, 2020; Zhou et al, 2022). Fibroblasts in the heart and lung activate inflammatory cytokine expression and recruit immune cells in acute injury or infection (Boyd et al, 2020; Choi et al, 2004; Kawaguchi et al, 2011; Mouton et al, 2019; Yu et al, 2013). Dermal fibroblasts activate cytokine expression in response to toll-like receptor stimulation or treatment with inflammatory factors (Al-Rikabi et al, 2021; Kitanaka et al, 2019; Ploeger et al, 2013; Yao et al, 2015). Although relatively few studies investigate dermal fibroblasts in vivo during the early stages of wound healing, inflammatory fibroblasts have been observed 1 day after wounding (Hu et al, 2023), and fibroblasts at 4 days after wounding are enriched for expression of immunomodulatory cytokines and chemokines (Almet et al, 2024). In addition, an inflammatory phenotype was recently described as an essential step in the development of wound myofibroblasts (Correa-Gallegos et al, 2023). In this study, we confirm that fibroblasts are potent modulators of inflammation in the earliest stages of the wound-healing response, upregulating a myriad of factors with functional importance in wound healing (Figure 6). These include factors such as *Ccl2*, *Ccl7*, and *Il33*, which are enriched in fibroblasts 4 days after wounding (Almet et al, 2024; Haensel et al, 2020). This finding was emphasized by the fact that fibroblast-induced macrophage migration was comparable with that of KCs and monocytes/macrophages, 2 well-studied cellular sources of chemotactic factors (Guenin-Mace et al, 2023; Pang et al, 2022; Ridiandries et al, 2018; Roupé et al, 2010; Villarreal-Ponce et al, 2020). Although we focused predominantly on the role of fibroblasts in macrophage/monocyte recruitment, we also observed upregulation of factors involved in neutrophil chemotaxis (*Cxcl2*) (De Filippo et al, 2013; Sawant et al, 2021) and macrophage polarization (*Il33* and *Il34*) (He et al, 2017; Zhuang et al, 2023) after injury. Given that fibroblasts may regulate neutrophil migration and macrophage polarization in other inflammatory contexts (Ferrer et al, 2017; Le Fournis et al, 2021; Williams et al, 2021; Witowski et al, 2009), these findings suggest that fibroblasts perform multiple conserved roles in innate immune cell inflammation across tissues.

In the skin, robust myeloid cell infiltration, particularly of macrophages, is essential for successful repair, as evidenced by the delayed and impaired healing observed when early immune cell infiltration is impeded (Boniakowski et al, 2018; Cooper et al, 2024; Goren et al, 2009; Lucas et al, 2010; Mirza et al, 2009; Sawaya et al, 2020; Shook et al, 2016; Willenborg et al, 2012; Wood et al, 2014). Although many chemokines participate in generating a chemotactic gradient to draw immune cells to the wound, CCL2 has been consistently identified as a key factor for monocytes/macrophages in this process (Willenborg et al, 2012; Wood et al, 2014). *Ccl2* expression was previously observed in dermal fibroblasts after injury (Correa-Gallegos et al, 2023; Shook et al, 2018), and CCL signaling has been identified as a key communication axis between fibroblasts and immune cells after injury (Almet et al, 2024). In this study, we functionally validated that fibroblast-derived CCL2 is essential for robust wound monocyte/macrophage numbers early after injury and timely wound closure and repopulation. Given that CCL2 also drives monocyte/macrophage recruitment after injury in other tissues such as the heart, lungs, and skeletal muscle (Dewald et al, 2005; Lu et al, 2011; Pappritz et al, 2018; Suresh et al, 2012), it will be interesting to see whether fibroblasts are also an important source of this chemokine in other injury settings and expand our understanding of critical fibroblast–macrophage communication across tissues and conditions (Zhou et al, 2022).

Skin fibroblasts are highly heterogeneous, with differences in genetic lineage or spatial location in the skin often underscoring the unique functions of different subsets (Abbasi et al, 2020; Correa-Gallegos et al, 2023; Driskell et al, 2013; Foster et al, 2021; Guerrero-Juarez et al, 2019; Korosec et al, 2019; Leavitt et al, 2020; Phan et al, 2020; Rinkevich et al, 2015). We observed upregulation of proinflammatory factors in fibroblasts, particularly those expressing SCA1 (Driskell et al, 2013; Lichtenberger et al, 2016; Rognoni et al, 2016; Sun et al, 2023). Although this study lacks the detection of SCA1+ fibroblasts with enriched chemokine production in tissue sections, our spatial analysis demonstrates strong expression of *Ccl2*, *Ccl7*, and *Il33* in fibroblasts residing in the superficial fascia. This echoes the finding that fascia-resident fibroblasts exhibit a proinflammatory profile after inflammation (Almet et al, 2024; Correa-Gallegos et al, 2023). SCA1+ fibroblasts were previously predicted to influence inflammation and chemotaxis on the basis of their gene expression in uninjured skin (Philippeos et al, 2018). These fibroblasts share markers of human fibroblasts found in the deep dermis, which respond robustly to inflammatory cues after isolation from healthy skin (Korosec et al, 2019; Philippeos et al, 2018). Moreover, SCA1 also delineates 2 major fibroblast populations in the heart, and SCA1+ cardiac fibroblasts are enriched for expression of *Ccl2* and other chemokines compared with SCA1− fibroblasts after heart injury (Chen et al, 2018; Farbehi et al, 2019). This implies that subset-specific inflammatory fibroblast signaling may be conserved across tissues. Why these subsets are enriched for this function requires further investigation.

Dysregulated inflammatory signaling from fibroblasts has been associated with various pathologies in the skin and other organs. In diabetic and aged skin wounds, fibroblasts express decreased levels of immune cell chemoattractants, yet maintain excessive expression of other factors that drive chronic inflammation and poor healing (Al-Rikabi et al, 2021; Januszyk et al, 2020; Mahmoudi et al, 2019; Vu et al, 2022; Wall et al, 2008). Despite this, fibroblasts enriched for the expression of inflammation-associated genes are associated with successful healing in diabetic foot ulcers (Choi et al, 2024; Theocharidis et al, 2022). Similarly, although our data highlight the importance of fibroblast inflammatory gene expression and CCL2 production for timely healing, sustained inflammatory signaling and even CCL2 production from fibroblasts have been associated with fibrosis and scar formation in the skin and other organs (Bhattacharyya et al, 2018; Burke et al, 2010; Chen et al, 2018; Correa-Gallegos et al, 2023; Foster et al, 2021; Jiang et al, 2020; Paish et al, 2018; Sinha et al, 2022; Wong et al, 2011; Wu et al, 2014; Zhang et al, 2012). Our findings emphasize the importance of inflammatory fibroblast subsets to guide appropriate immune responses early after injury (Liu et al, 2024). Identifying factors that initiate, suppress, or prolong this inflammatory state in fibroblasts will be fundamental to addressing healing pathologies associated with insufficient or excessive inflammation.

## MATERIALS AND METHODS

### Animals

Animal maintenance and experiments were conducted under the guidance of George Washington University’s Institutional Animal Care and Use Committee. C57/Bl6 mice were purchased from Jackson Laboratories (strain 000664). *Pdgfra*CreER; *Ccl2*^fl/fl^ mice were generated by crossing B6.129S-*Pdgfra*tm1.1(cre/ERT2)Blh/J (Jackson Laboratories, strain 032770) with B6.Cg-*Ccl2*^*tm1.1Pame*^/J (Jackson Laboratories, strain 016849). *Pdgfra*CreER; mT/mG mice were generated by crossing B6.129S-*Pdgfra*tm1.1(cre/ERT2)Blh/J (Jackson Laboratories, strain 032770) with *Gt(ROSA)26Sor*^*tm4(ACTB-tdTomato,-EGFP)Luo*^/J (Jackson Laboratories, strain 007576). Tamoxifen was administered to *Pdgfra*CreER; *Ccl2* ^fl/fl^ or *Pdgfra*CreER; mT/mG mice topically to shaved back skin (2 times applications of 100 μl of 5 mg/ml in ethanol, Sigma-Aldrich, T5648) for all experiments except those in Figure 4d–g, Figure 4i, and Supplementary Figure S5c–h. For these experiments, tamoxifen was administered by intraperitoneal injection (2 times 100 μl injections of 30 mg/ml in sesame oil, Sigma-Aldrich, S3547-250ML). Mice were euthanized with carbon dioxide and cervical dislocation. All experimental procedures were approved in accordance with George Washington University’s Institutional Animal Care and Use Committee. Male mice were used for all experiments unless otherwise noted in figure legends.

### Dorsal skin excision

Male and female mice in the telogen phase of the hair cycle were used (aged 7–10 weeks). For wounding experiments, mice were anesthetized with 4% isoflurane and inflicted with 2 or 4 excisional wounds 4–6 mm apart on shaved back skin by a 4-mm biopsy punch (Miltex).

### Isolation of single nuclei for RNA sequencing

Methods were adapted from previous reports (Li and Humphreys, 2022; Habib et al, 2017). To make nuclei lysis buffer 0 (NLB0), 1 cOmplete ULTRA tablet (Roche, 05892791001) was dissolved per 10 ml Nuclei EZ Lysis Buffer (Sigma-Aldrich, Nuc-101). To make NLB1, 10 μl RNasin Plus ribonuclease inhibitor (Promega, N2615) and 10 μl SUPERaseIN RNase Inhibitor (Invitrogen, AM2694) were added per 4 ml NLB0. To make NLB2, 4 μl RNasin Plus ribonuclease inhibitor and 4 μl SUPERaseIN Rnase Inhibitor were added per 4 ml NLB0. All steps were performed on ice. Skin samples were obtained from male C57BL/6 mice. Briefly, unwounded skin and wounds 24 hours after wounding (wound center and ≤2 mm of the wound periphery) were collected and minced to approximately 1 mm^2^ in NLB1. Minced tissue was transferred to KONTES dounce tissue grinders (Kimble Chase KT885300–0002) with an additional 1 ml of NLB1. The loose pestle (A) was applied 22 times before passage through 200-μm pluriStrainer (pluriSelect 43–50200). The tight pestle (B) was applied 12 times, and then the homogenate was incubated for 5 minutes with an additional 2 ml NLB1. Then, the isolated nuclei were passed through a 40-μm pluriStrainer (pluriSelect 43–50040), centrifuged at 500*g* for 5 minutes at 4 °C, and resuspended in 4 ml NLB2. Resuspended samples were incubated on ice for 5 minutes and then centrifuged. Samples were resuspended in nuclei suspension buffer (1 μl RNasin Plus/ml 2% BSA in Dulbecco’s PBS). Nuclei were washed by centrifugation and resuspension 4 times before a final resuspension in 250 μl nuclei suspension buffer and filtration through a 5-μm strainer. A total of 50 μl were taken for assessment and counting of nuclei, and the remainder were diluted according to 10X protocol targeting 10,000 nuclei per sample.

### Construction of 10X Genomic single-cell 3^’^ RNA-sequencing libraries and sequencing

Immediately after isolation, 2 wounded and 2 uninjured samples were loaded into 10X genomics Next GEM G chips (10X Genomics, 2000177) with Next GEM Single Cell 3’ Gel Beads, version 3.1 (10X Genomics, 2000164), for subsequent lysis, cDNA synthesis, and amplification. Library preparation was performed using Single Index Kit T Set A (10X Genomics, 2000240). Samples were balanced and sequenced by Genewiz (now Azenta), with the aim of 50,000 reads per cell. All 4 samples were pooled in 1 lane on an Illumina NovaSeq 6000 instrument, which averages 2 billion reads per lane. Data are available at Gene Expression Omnibus (GSE265996) (reviewer token: ovwriiaqdlkztur).

### Quantification and integration of snRNA-seq data

Data preprocessing and analysis were performed using Cell Ranger 5.0.1 (10X Genomics) (Zheng et al, 2017). Briefly, Cell Ranger’s count utility was used to align the raw sequencing reads to mm10 and to generate feature-barcode matrices for downstream analyses. To accommodate the characteristics of nuclear RNA, which includes a significant proportion of unspliced pre-mRNA, the “–include-introns” flag was used. Quality metrics, such as the total number of detected genes per nucleus, the proportion of reads mapping to the nuclear genome, and the distribution of reads across intronic and exonic regions, were assessed to filter out low-quality nuclei.

Downstream quality control and data integration were performed in R using Seurat (Satija et al, 2015; Stuart et al, 2019). Samples were filtered on the basis of the number of features, RNA count, and percentage of mitochondrial genes per nucleus, following established quality-control methods (Luecken and Theis, 2019; Osorio and Cai, 2021). Owing to variations in sample quality, filtering parameters were determined for each sample (Luecken and Theis, 2019). For unwounded samples #1 and #2, the nFeature_RNA > 200, and nFeature_RNA < 2500 with nCount_RNA < 8000 and the percent.mt < 1. For 1-day-postwounding sample #1, the nFeature_RNA > 200, and nFeature_RNA < 3000 with nCount_RNA < 6000 and the percent.mt < 1.5. For 1-day-postwounding sample #2, the nFeature_RNA > 200, and nFeature_RNA < 1750 with nCount_RNA < 2250 and the percent.mt < 1. Data normalization was performed using sctransform, version 0.4.1 (Hafemeister and Satija, 2019). After normalization, integration was performed using Seurat’s FindIntegrationAnchors() and IntegrateData() functions using the default setting of 30 canonical correlation analysis dimensions (Hao et al, 2021). Twenty-one cell clusters were subsequently identified with Seurat’s standard RunPCA(), FindClusters(), and FindNeighbors() functions, using the first 30 principle components and a resolution of 0.8 (Xu and Su, 2015). Seurat’s FindAllMarkers() function was used to identify genes associated with each cluster.

### Gene expression, cell communication, and pathway enrichment analysis

Average gene expression in the uninjured and wounded conditions was calculated after normalization of the RNA counts using Seurat’s NormalizeData() function, using LogNormalize as the normalization method. To analyze potential communication networks between groups of cells, we used CellChat, version 2.1.2 (Jin et al, 2024, 2021), on the normalized RNA count data. Owing to the shallower sequencing depth of the dataset, we projected the gene expression data onto a protein–protein interaction network using the project-Data() function and used the “truncated mean” setting with trim = 0.01 in the computeCommunProb() function. To measure differences in gene expression between the unwounded and injured conditions in the snRNA-seq dataset, we employed Tweedieverse, version 0.0.1 (Mallick et al, 2022), targeting a false discovery rate of 0.05 and using the Benjaminie–Hochberg procedure to report significance. Significant changes in gene expression are reported as the Tweedieverse effect size. Pathway enrichment analysis to predict biofunctions associated with changes in gene expression after injury was performed using Ingenuity Pathway Analysis (Krämer et al, 2014). For Tweedieverse analysis comparing uninjured with 1-day-postwounding samples within individual cell clusters, only genes with an average expression level ≥2 in at least 1 condition in the total dataset and a significant Tweedieverse effect size were reported.

### Flow cytometry and sorting

Mouse back skins or wounds (wound bed and ≤2 mm of the wound periphery) were excised and minced. Samples were digested in RPMI (Gibco, 61870–036) with 0.25 mg/ml Liberase TM (Roche, 05401127001), 1 mM sodium pyruvate (Gibco, 11360070), 1% antibiotic-antimycotic (Gibco, 15240062), 50 U/ml deoxyribonuclease I (Worthington, LS002006), 2% MEM nonessential amino acids (Gibco, 11140050), and 25 mM 4-(2-hydroxyethyl)piperazine-1-ethane-sulfonic acid buffer (Gibco, 15630080). Samples were incubated in a shaker incubator at 37 °C for 90 minutes to release single cells. Tissue samples were consecutively filtered through 70-μm and 40-μm strainers. Single-cell suspensions from tissue digestion or epidermal isolation were resuspended in FACS staining buffer (1% BSA in PBS [Gibco, 14190–250] with 2 nM EDTA and 25 U/ml deoxyribonuclease I) and immunostained for 20 minutes on ice.

To assess mesenchymal heterogeneity, cells were stained with the following antibodies: CD45-allophycocyanin (APC)-Cy7 (BioLegend, 1:1000), CD31-APC-Fire-750 (BioLegend, 1:500), CD29-Alexa700 (BioLegend, 1:500), EpCAM-APC-Fire-750 (BioLegend, 1:500), and SCA1 (LY6A/E)-BV650 (BioLegend, 1:1500). For myeloid cell immunophenotyping, cells were stained with the following antibodies: CD45-APC-Cy7 (BioLegend, 1:1000), CD11b-Alexa700 (BioLegend, 1:500), F4/80-eFluor450 (eBioscience, 1:200), and Ly6G-BV785 (BioLegend, 1:200) or -PE-Cy7 (BioLegend, 1:500). Wound monocytes were defined as CD45+, CD11b+, F4/80−, Ly6G− cells. Macrophages were defined as CD45+, CD11b+, F4/80+, Ly6G− cells. Neutrophils were defined as CD45+, CD11b+, F4/80−, Ly6G+ cells. For experiments where fibroblasts and the monocyte/macrophage population were isolated from the same wounds, cells were stained with CD45-FITC (BioLegend, 1:1000), CD31-APC-Fire-750 (BioLegend, 1:500), CD11b-Alexa700 (BioLegend, 1:500), EpCAM-APC-Fire-750 (BioLegend, 1:500), Ly6G-PE-Cy7 (BioLegend, 1:500), and SCA1-BV650 (BioLegend, 1:1500). Wound monocyte/macrophages were defined as CD45+CD11b+ Ly6G− cells.

For KC isolation, total back skin or 1–2 mm of skin surrounding wounds was harvested from the backs of the mice. Fat was scraped from the bottom of the skin using a blunt scalpel. The skin samples were placed dermis side down in a sterile culture dish and rinsed in PBS without calcium ion or magnesium ion (Gibco, 14190–250). Samples were suspended in 0.25% Trypsin-EDTA (Gibco, 25200072) and incubated for 75 minutes at 37 °C. After incubation, hair was scraped from the skin using curved forceps and the scalpel. Samples were broken down with the scalpel and triturated with a 25-ml pipet for ~50 seconds. Samples were filtered through 70-μm and 40-μm strainers, and cells were stained with CD45-APC-Cy7 (BioLegend, 1:1000) and EpCAM-APC (BioLegend, 1:100) to isolate CD45−, EpCAM+ cells.

To exclude dead cells, samples were incubated with Zombie Aqua (BioLegend, 423102, 1:1000) at room temperature for 20 minutes before antibody staining, or Sytox Blue or Sytox Green (Invitrogen, S34857 or S34860, 1:1000) was added before analysis. Analysis was performed on a Celesta (BD Biosciences) or CytoFLEX S (Beckman Coulter Life Sciences), and sorting was performed on a BD Influx (BD Biosciences) or a Sony SH800 (SONY). Cells were sorted into 10% fetal bovine serum (FBS) (Gibco, A4766801) and 1% antibiotic-antimycotic (Gibco, 15240062) in DMEM (ATCC, 30–2002), and flow cytometry analysis was performed using FlowJo Software (FlowJo).

Additional information on antibodies used for flow cytometry can be found in Supplementary Table S1.

### RT-qPCR

FACS-purified cells from uninjured skin or wound beds were lysed in TRIzol (Invitrogen, 15596018); RNA was isolated from the aqueous phase and purified using the RNeasy Micro kit (Qiagen, 74004). The Superscript IV kit (Invitrogen, 18-090-200) was used to generate cDNA proportionally to the isolated RNA. RT-qPCR was performed using PowerTrack SYBR Green master mix (Applied Biosystems, A46111) run on a Bio-Rad CFX384 with a C1000 thermocycler. Gene-specific primer sequences are listed in Supplementary Table S2. All reported values reflect the levels of target mRNA normalized to β-actin. mRNA levels are the average of biological samples (n ≥ 3) calculated from technical qPCR triplicates.

### ELISA

Skin wounds were digested and stained for flow cytometry as described earlier, and cell populations were sorted on a BD Influx. For data shown in Figure 2f and g, undiluted cell conditioned media from the migration assays (described earlier) was used in a CCL2 ELISA (R&D Systems, MJE00B). For Figure 3f–h, cells were plated in a 24-well tissue culture plate (Corning, COSTAR 3526) at a density of at least 36,000 cells/ml in DMEM with 10% FBS and 1% antibiotic-antimycotic and incubated at 37 °C for 2.5 hours. Cell supernatant was harvested after incubation. ELISA kits were used to measure the concentration of CCL2 (R&D Systems, MJE00B), CCL7 (Invitrogen, BMS6006INST), and IL-33 (R&D Systems, M3300). Briefly, supernatant (2 technical replicates per sample) was incubated on the provided plate, along with a set of standard dilutions. After washing the wells, conjugate specific to the analyte was added, followed by further incubation, washing, and addition of substrate. The optical density of each well was determined using a BioTek Synergy H1 Hybrid Reader set to 450 nm. Cell supernatant was either diluted prior to the ELISA, or the detection values were normalized to the lowest seeding density if the number of fibroblasts seeded differed between biological replicates.

### Bone marrow isolation and macrophage differentiation

The femur and tibia were isolated from both hind limbs. After cleaning, the bones were cut at both ends, and bone marrow was flushed out with sterile RPMI and a 25-gauge needle. Red blood cells were lysed with 2 ml of ACK lysis buffer (150 mM ammonium chloride, 10 mM potassium bicarbonate, 0.1 mM Na_2_EDTA). After buffer neutralization with RPMI plus 5% FBS, cells were passed through a 70-μm filter. Cells were plated at a density of 3 × 10^5^ cells/ml in 10 ml of RPMI plus 30% L929 conditioned media, 10% FBS, and 1% antibiotic-antimycotic in a 10-cm petri dish. On day 3 or day 4 after plating, an additional 10 ml of this media was added to the plates. BMDMs were used for experiments on day 6 or day 7. L929 cells (MilliporeSigma, 85011425–1VL) were cultured, and conditioned media was harvested as previously described (Weischenfeldt and Porse, 2008).

### Transwell migration assay

#### BMDM migration in response to fibroblasts, myeloid cells, and KCs.

Twenty-four hours prior to the migration assay, BMDMs were serum starved in RPMI with 1% antibiotic-antimycotic. Serumstarved BMDMs were lifted from the plate with a cell scraper in cold PBS and resuspended in RPMI with 1% FBS and 1% antibiotic-antimycotic at 1 × 10^6^ cells/ml. Fibroblasts (CD45–CD31–EpCAM−), myeloid cells (CD45+CD11b+Ly6G−), and KCs (CD45−EpCAM+) were sorted from uninjured skin or wound samples 1.5 days after wounding after skin digestion (fibroblasts and macrophages) or epidermal isolation (KCs) as described earlier. These primary cells were plated at a density of ~80,000 cells/ml in RPMI with 1% FBS and 1% antibiotic-antimycotic immediately after sorting in 24-well tissue culture plates. A total of 1 × 10^5^ BMDMs were added to a transwell insert with 8-μm pore diameter (MilliporeSigma, PTEP24H48) into the wells with the primary cells. As a positive control, BMDMs treated overnight with 100 ng/ml lipopolysaccharide (Sigma-Aldrich, L4391–1MG) were plated in the lower chamber. The plates were incubated at 37 °C with 5% carbon dioxide overnight. Media from the wells was harvested after incubation and spun down to remove any debris before storage at −80 °C until use.

#### Comparing myeloid cell migration between PdgfraCreERD; and PdgfraCreERL; Ccl2^fl/fl^ mice.

SCA1+ fibroblasts and monocytes/macrophages were sorted from the same 1.5-day wounds. SCA1+ fibroblasts were plated at a density of 36,000 cells/ml in the bottom of a 24-well tissue culture transwell insert plate (Corning, COSTAR 3464, 8 μm pore diameter) in DMEM. A total of 100 μl of wound-matched monocytes/macrophages (density of 372,000/ml in DMEM) were seeded in the transwell insert. Transwell cultures were incubated overnight at 37 °C with 5% carbon dioxide.

For all migration assays, after incubation, transwells were removed and stained in crystal violet (Sigma-Aldrich, V5265–500ML, 0.2% in methanol) for 10 minutes. Transwells were rinsed in water, and the inside was gently cleaned with a cotton swab. Inserts were left to dry for at least 24 hours. For the BMDM migration assay, 5–8 representative images from each transwell were taken for quantification. For the SCA1+/wound monocyte–macrophage assay, ×20 tile images of each edge of the membrane as well as a region from the membrane center were taken for each insert. For both experiments, the number of migrated cells was calculated using QuPath software, version 0.3.2 (Bankhead et al, 2017). QuPath’s pixel classifier tool was used to train the software to recognize areas of positive crystal violet staining, which were then quantified per image. For the BMDM migration assay, data were reported as the average number of BMDMs per field of view for each transwell. For the SCA1+/wound monocyte–macrophage assay, the area analyzed was the same for each transwell, and the total number of migrated cells was reported.

### In situ hybridization for RNA detection (RNAscope)

Uninjured skin and 1.5-day wounds with 1.5 mm of nonwound skin cut around the edge from male C57 mice were dissected, embedded in Scigen Tissue-Plus Optimal Cutting Temperature Compound (Thermo Fisher Scientific, 23-730-571), frozen, and cryosectioned into 16-μm sections on a Micron HM305 E Cryostat within 1 week of harvest. Tissue sections were stained using the RNAscope Multiplex Fluorescent Reagent Kit v2 with TSA Vivid Dyes (ACDBio, 323270), according to manufacturer instructions. Briefly, samples were fixed at room temperature in 10% neutral buffered formalin, dehydrated in ethanol, and treated with protease IV from the kit (322336). Sections were treated with probes targeting *Pdgfra* (480661-C2) and *Ccl2* (311791-C3), *Pdgfra* and *Ccl7* (446821-C3), or *Pdgfra* and *Il33* (400591-C3), followed by kit amplifiers. *Pdgfra* signal was detected with TSA Vivid Fluorophore 650 (1:1300 dilution, PN 323273), and *Ccl2/Ccl7/Il33* signal was detected with TSA Vivid Fluorophore 570 (1: 1300 dilution, PN 323272). Sections were treated with the provided DAPI solution (323108) and mounted in ProLong Gold antifade reagent (Invitrogen, P36934).

### Immunofluorescence staining

Naïve mouse back skin or wound beds were dissected, embedded in Scigen Tissue-Plus Optimal Cutting Temperature compound (Thermo Fisher Scientific, 23-730-571), frozen, and cryosectioned into 14–16-μm sections on a Micron HM305 E Cryostat. Wounds were sectioned in their entirety and sequentially distributed across multiple slides to enable identification of the wound center and multi-parameter analysis of the same wound, as previously detailed (Haas et al, 2021). Frozen tissue sections were fixed in 4% formaldehyde and immunostained with the following antibodies: CCR2 (BioLegend, 1:100), CD31 (BD Pharmingen, 1:50), ER-TR7 (Abcam, 1:500), F4/80 (Abcam, 1:200), GFP (Abcam, 1:1000), ITGA6 (R&D Systems, 1:300), Perilipin-1 (Abcam, 1:500), pH3 (Abcam, 1:500), and smooth muscle actin (Abcam, 1:400). Slides were mounted in ProLong Gold Antifade mountant with DAPI (Invitrogen, P36935) for nuclear staining. Additional information on antibodies used for immunofluorescence can be found in Supplementary Table S1.

### Imaging

All imaging was performed using a Zeiss AxioImager M2. Brightfield images of the crystal violet–stained transwells were taken with an Axiocam 305 color camera (Zeiss). Images of immunofluorescence or RNA in situ hybridization were acquired with an Orca camera (Hamamatsu). Tile scans of wound beds and the wound periphery were acquired using ZEN software, version 3.0 (Zeiss). For wounds, the 2 or 3 central most sections were imaged.

### Image analysis

Analyses and quantification were performed on the 2–3 most central sections of the wound. For analyses of wound beds, values from the different sections were averaged as a single wound bed value. Results were reported as single wounds or wound edges as described in figure legends and previously described (Schmidt and Horsley, 2013; Shook et al, 2020, 2018). For all analyses, wounds were taken from at least 3 different mice per group.

For F4/80 and CCR2 analysis in the wound center 1.5 days after wounding, nuclear signals associated with F4/80 and CCR2 staining were hand counted, and the wound bed area was quantified in Adobe Photoshop. For F4/80 analysis at the wound periphery, wound edges were cropped to 800 μm in width from the edge of the wound and normalized to the same height in pixels. For consistency, 150–200 pixels of fascia below the panniculus carnosus were included in each image. Wound-edge images were excluded if they differed significantly in the orientation of the skin layers compared with the rest of the set. F4/80+ cells in these images were identified using MATLAB 2023b as previously described (Cooper et al, 2024). Briefly, using the regionprops() function, areas of staining in the images were called F4/80+ cells if there was at least 75% overlap of the nuclear periphery and F4/80 signals. For spatial analysis, images were divided into 20 bins along the superficial–deep and proximale–distal axes, and the number of cells in each bin was calculated. Total F4/80+ cells in each edge and their pixel coordinates on the image are also reported. To generate heat maps of F4/80 spatial distribution at the wound edge, the pixel coordinates from the cells in each image from a group were graphed as a rasterized, 2-dimensional density plot in R, version 4.3.1. All woundedge images were analyzed individually and not averaged per wound.

For RNAscope analysis, unwounded images were cropped to 750 μm in length. The 1.5-day wounds were analyzed at the wound periphery and cropped to 750 μm from the wound edge with the same height. Images were analyzed using QuPath, version 0.3.2 (Bankhead et al, 2017). The CellDetection function was used to identify cells on the basis of DAPI staining, followed by the SubcellularDetection function to identify cells with positive staining for *Pdgfra*, *Ccl2*, *Ccl7*, or *Il33*. The pixel coordinates of *Pdgfra*+*Ccl2*+, *Pdgfra*+*Ccl7*+, or *Pdgfra*+*Il33*+ cells on the images were subsequently obtained. Spatial heat maps were generated as described earlier in R, version 4.3.1.

ImageJ (FIJI) was used to measure the total wound width and the length not covered by ITGA6 to calculate re-epithelialization, and the length of the wound epithelium was directly measured in ImageJ as previously described (Yan et al, 2022). Wounds with continuous ITGA6+ epithelium were defined as closed, and wounds with discontinuous ITGA6+ epithelium were defined as open.

The Color Range feature of Adobe Photoshop was used to identify CD31+ pixels, ER-TR7+ pixels, and total wound bed pixels to determine percentage revascularization and fibroblast infiltration, respectively. Spatial analysis of CD31 was performed using ImageJ (FIJI) after cropping and blacking out nonwound areas in Photoshop. Proliferation analysis of pH3 staining was performed by identifying pH3+ cells in ImageJ (FIJI) and is reported as a function of the number of pH3+ cells in the region of interest divided by the total area of that region in mm^2^.

### Statistics

All statistics were performed using GraphPad Prism, version 9. Unpaired, 2-tailed Student *t*-tests were used for comparisons between 2 groups, and 1-way ANOVAs with Tukey’s multiple comparisons test were used for comparisons between multiple groups. For all statistical tests, *P* < .05 was considered significant.

## Supplementary Material

1

## Data Availability

Datasets related to this article can be found at https://www.ncbi.nlm.nih.gov/geo/query/acc.cgi?acc=GSE265996, hosted at the National Center for Biotechnology Information Gene Expression Omnibus database GSE265996.

## Abbreviations:

APC: allophycocyanin
BMDM: bone marrow–derived macrophage
FBcKO: fibroblast conditional knockout
FBS: fetal bovine serum
KC: keratinocyte
pH3: phospho-histone H3
snRNA-seq: single-nuclei RNA-sequencing
