# Supplementary material for: Fibroblast-Mediated Macrophage Recruitment Supports Acute Wound Healing
Source: J Invest Dermatol. Author manuscript; Available in PMC 2025 Jul 1. (PMC12095619; doi:10.1016/j.jid.2024.10.609)
Supplement: 1 [file NIHMS2038024-supplement-1.pdf]

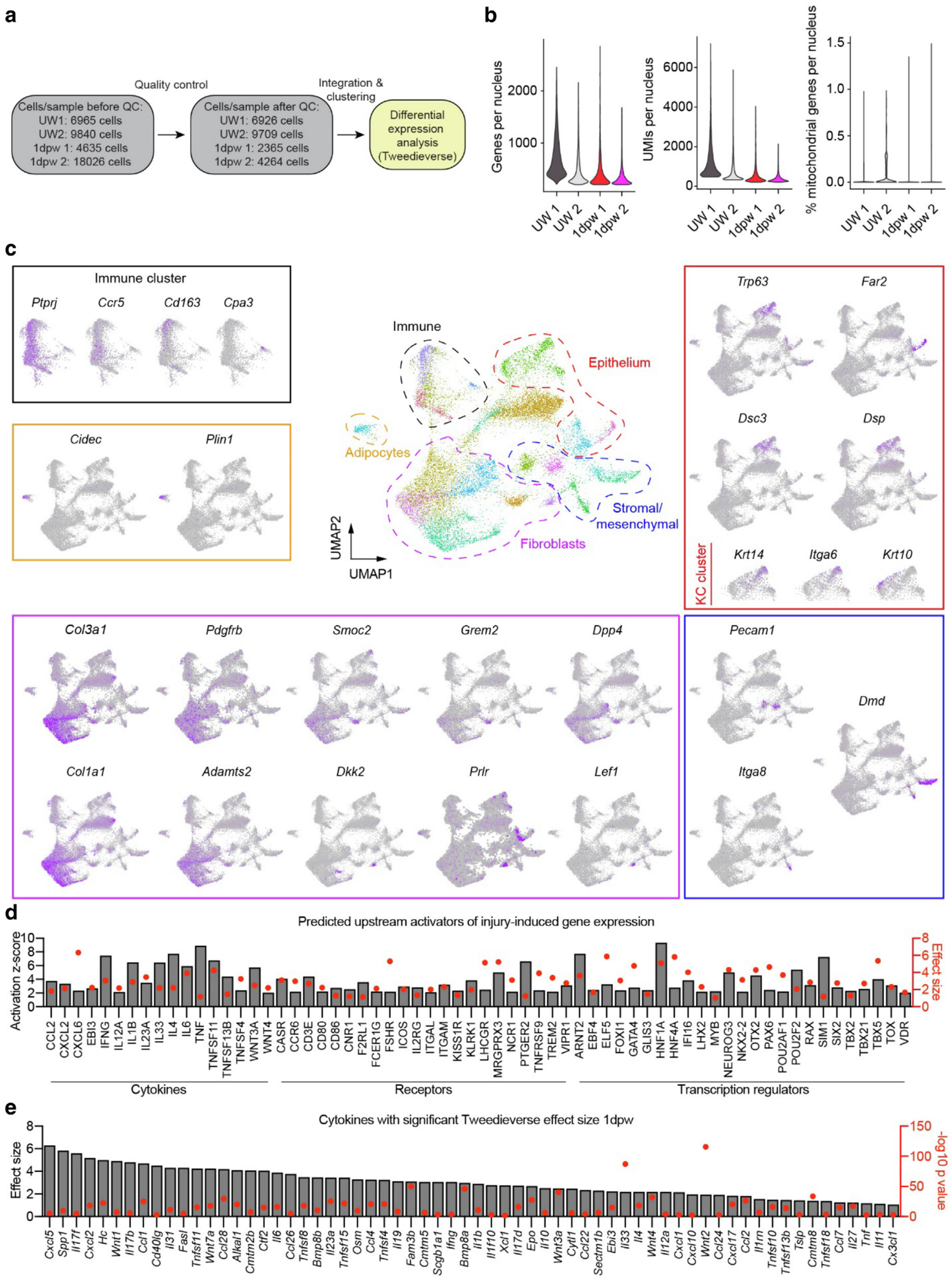

Supplementary Figure S1. Identification of cellular subsets and predicted regulators of injury-induced gene expression from snRNA-seq of skin. (**a**) Pipeline for snRNA-seq data analysis. (**b**) Number of genes identified per nucleus (left), number of UMIs per nucleus (center), and percentage of mitochondrial genes per nucleus (right) in each sample. (**c**) Feature plots for genes associated with specific cell populations. (**d**) Upstream activators of upregulated gene expression 1 dpw identified by Ingenuity Pathway Analysis. (**e**) Cytokines with significant effect size 1 dpw. dpw, day after wounding; snRNA-seq, single-nuclei RNA sequencing; UMAP, Uniform Manifold Approximation and Projection; UMI, unique molecular identifier.

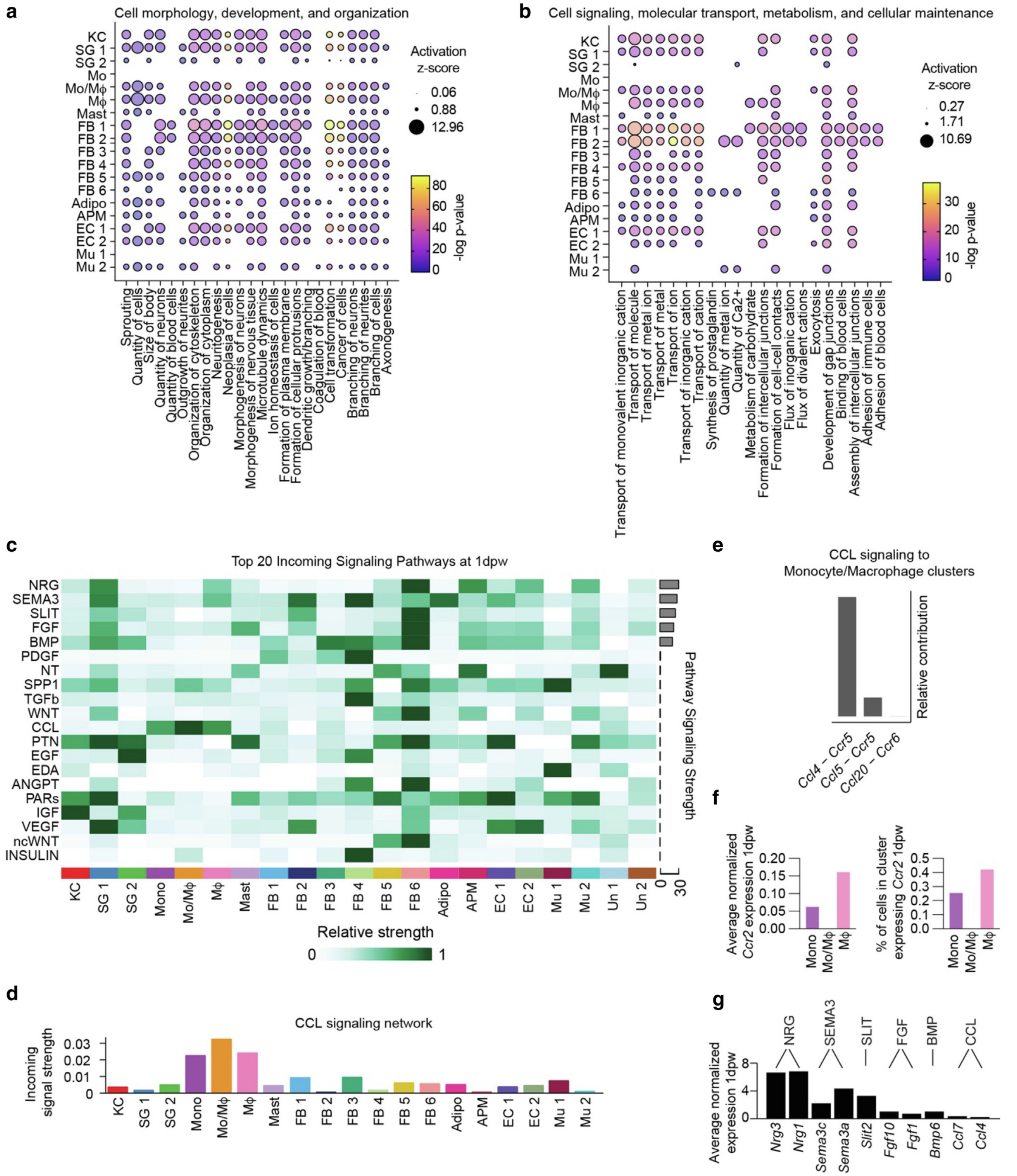

Supplementary Figure S2. Injury-induced changes in gene expression generate distinct and redundant predicted biological functions in cellular subsets. (**a, b**) Bubble matrices of biofunctions associated with upregulated gene expression in each cell cluster 1 dpw. Matrices are divided by terms relating to (**a**) cell morphology, development, and organization or (**b**) signaling, transport, metabolism, and cell maintenance. (**c**) Heat map of the top 20 predicted signaling pathways received by clusters 1 dpw. (**d**) Predicted incoming CCL signaling strength across individual cell clusters 1 dpw. (**e**) Predicted ligand−receptor interactions involved in CCL signaling to monocytes and macrophages 1 dpw. (**f**) Quantification of the average expression of *Ccr2* in monocyte/macrophage clusters and the percentage of cells with detectable levels of *Ccr2* transcripts. (**g**) Average expression levels of signaling factors in cells 1dpw. APM, arrector pili muscle; dpw, day after wounding; KC, keratinocyte.

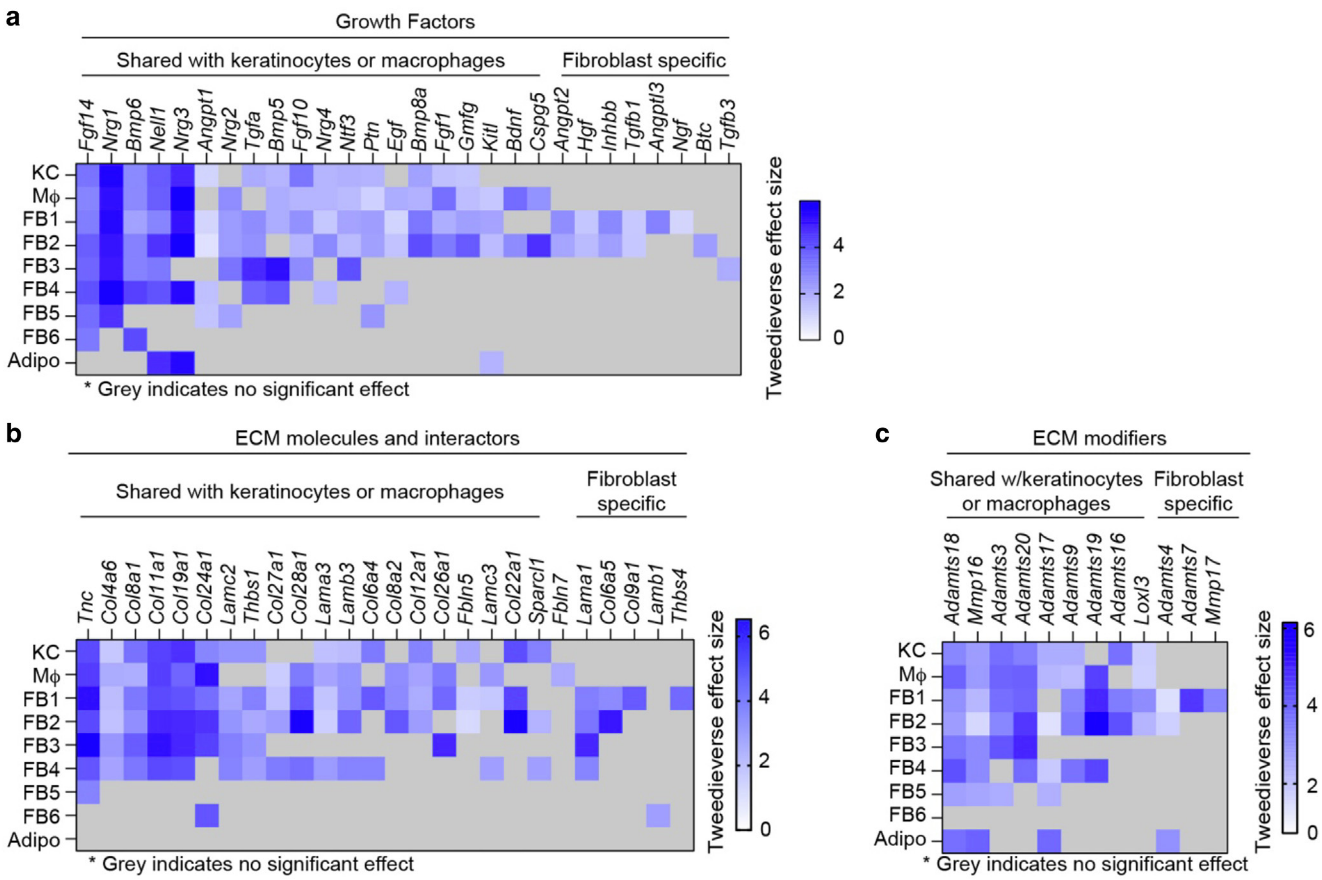

Supplementary Figure S3. Injury-induced distinct and redundant changes in gene expression of genes that can influence the wound environment. (**a**–**c**) Heatmaps of genes with significant Tweedieverse effect size 1 dpw and average expression >0.2 in the UW or 1 dpw condition. Tweedieverse data are categorized as (**a**) GFs, (**b**) extracellular matrix molecules and interactors, and (**c**) modifiers with an increased effect size in keratinocytes, macrophages, fibroblasts, and adipocytes 1 dpw. dpw, day after wounding; ECM, extracellular matrix; KC, keratinocyte; UW, unwounded.

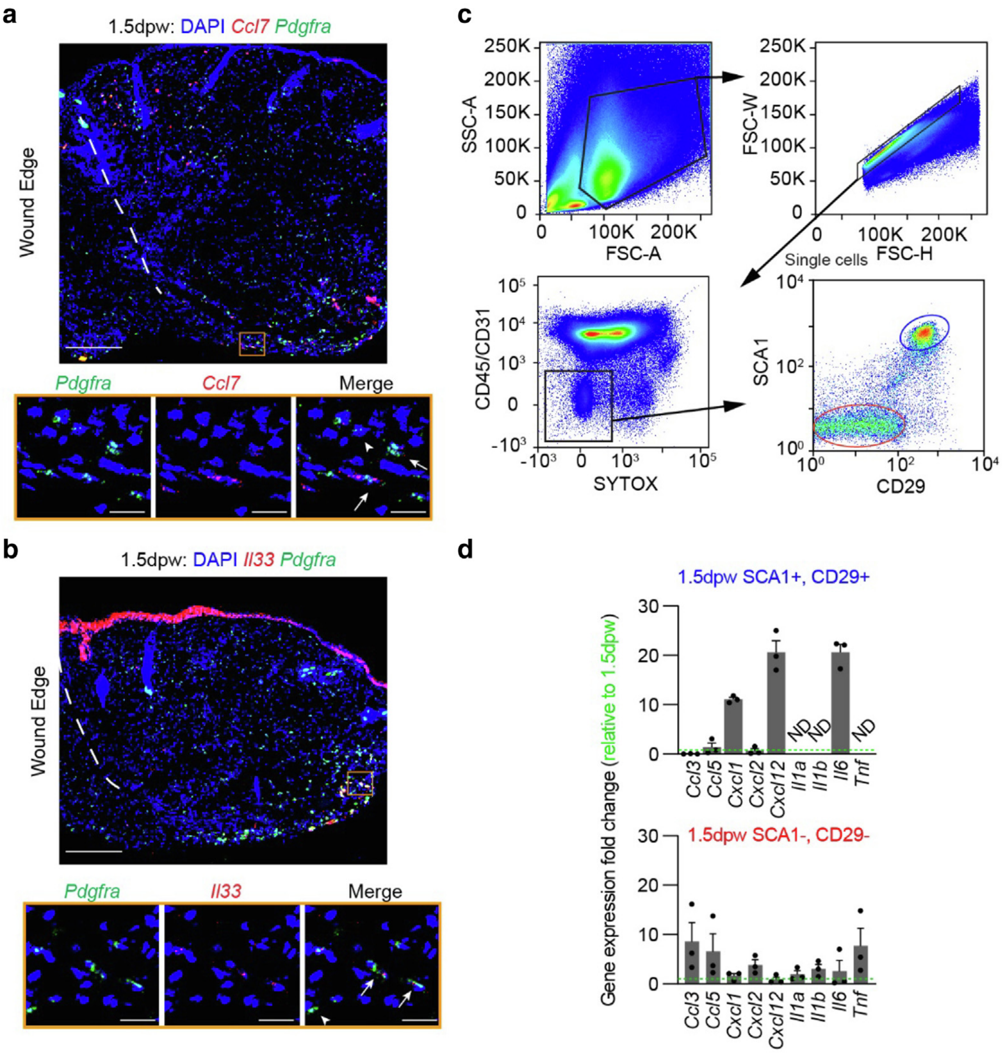

Supplementary Figure S4. Fibroblast populations exhibit distinct cytokine expression profiles after injury. (**a**) Representative composite images of RNAScope analysis of *Ccl7* (red) and *Pdgfra* (green) expression 1.5 dpw at the wound periphery. Bottom images correspond to the orange boxed area in the top image. Bars = 200 μm (top image) and 20 μm (bottom images). Dotted white line corresponds to the wound edge. Examples of double-positive (*Pdgfra*+, *Ccl7*+) and single-positive (*Pdgfra*+) cells are highlighted by arrows and arrowheads, respectively. (**b**) Representative composite images of RNAScope analysis of *Il33* (red) and *Pdgfra* (green) expression 1.5 dpw at the wound periphery. Bottom images correspond to the orange boxed area in the top image. Bars = 200 μm (top image) and 20 μm (bottom images). Dotted white line corresponds to the wound edge. Examples of double-positive (*Pdgfra*+, *Il33*+) and single-positive (*Pdgfra*+) cells are highlighted by arrows and arrowheads, respectively. (**c**) Gating strategy for flow cytometry analysis of mesenchymal populations in skin. (**d**) Comparison of cytokine gene expression in the live, CD45−, CD31−, SCA1+, CD29+ and live, CD45−, CD31−, SCA1−, CD29− populations isolated 1.5 dpw. Gene expression was measured with qPCR and is normalized to the expression level in 1.5 dpw wound beds. n = 3 mice per group. dpw, day after wounding.

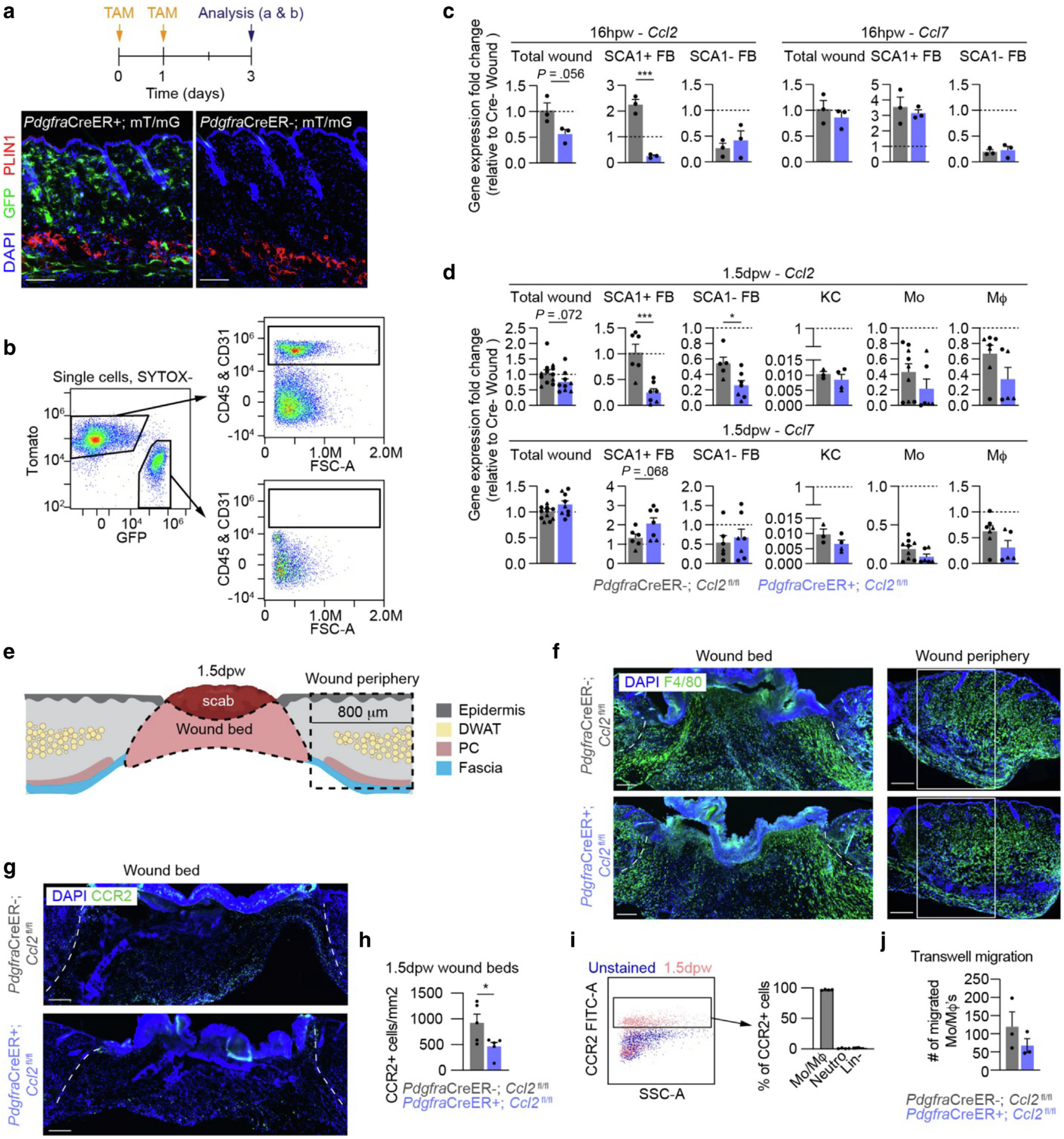

Supplementary Figure S5. FB-specific deletion of *Ccl2* alters chemokine expression and macrophage recruitment. (**a**) Images of tissue sections from *Pdgfra*CreER+; mT/mG and *Pdgfra*CreER−; mT/mG mice immunostained for GFP and PLIN1. Bars = 100 μm. (**b**) Flow cytometry plots of CD45 and CD31 levels in GFP and RFP populations in *Pdgfra*CreER+; mT/mG skin. (**c**) *Ccl2* and *Ccl7* expression 16 hpw in the total wound and sorted SCA1+ or SCA1− FBs. Data are normalized to the expression level of each gene in the total wound samples from control (*Pdgfra*CreER- ; *Ccl2*fl/fl) mice, indicated by the dashed line in each graph. n = 3 male mice per group. (**d**) *Ccl2* and *Ccl7* expression 1.5 dpw in the total wound and sorted SCA1+ and SCA1− FBs, keratinocytes (CD45−, EpCAM+), monocytes (CD31−, EpCAM−, CD45+, Ly6G−, F480−), and macrophages (CD31−, EpCAM−, CD45+, Ly6G−, F480+). Data are normalized to the expression level of each gene in the total wound samples from control (*Pdgfra*CreER−; *Ccl2*fl/fl) mice, indicated by the dashed line in each graph. n ≥ 3 mice per group. (**e**) Cartoon schematic showing the locations of images that were assessed. (**f**) F4/80-immunostained tissue sections and representative images from the wound bed and wound periphery in *Pdgfra*CreER−; *Ccl2*fl/fl and *Pdgfra*CreER+; *Ccl2*fl/fl mice. Boxed areas in the wound edge images delineate the region analyzed for F4/80 immunostaining. Bars = 250 μm. (**g**) Composite images of CCR2-immunostained tissue sections from 1.5 dpw wounds beds of *Pdgfra*CreER−; *Ccl2*fl/fl and *Pdgfra*CreER+; *Ccl2*fl/fl mice. Bars = 250 μm. (**h**) Quantification of CCR2+ cells in wound beds harvested 1.5 dpw. n ≥ 5 wounds per group from n = 4 mice per group. (**i**) Dot plot of CCR2 flow cytometry analysis in wounds 1.5 dpw and quantification of CCR2+ cells based on cell identities. Monocyte (Mo)/macrophage (Mϕ) (CD45+, CD11b+, LY6G−), neutrophils (CD45+, CD11b+, LY6G+), and total lineage- mesenchymal cells (CD31−, EpCAM−, CD45−) were analyzed. n = 4 mice. (**j**) Quantification of monocyte (Mo)/macrophage (Mϕ) (CD45+, CD11b+, LY6G−) migration through

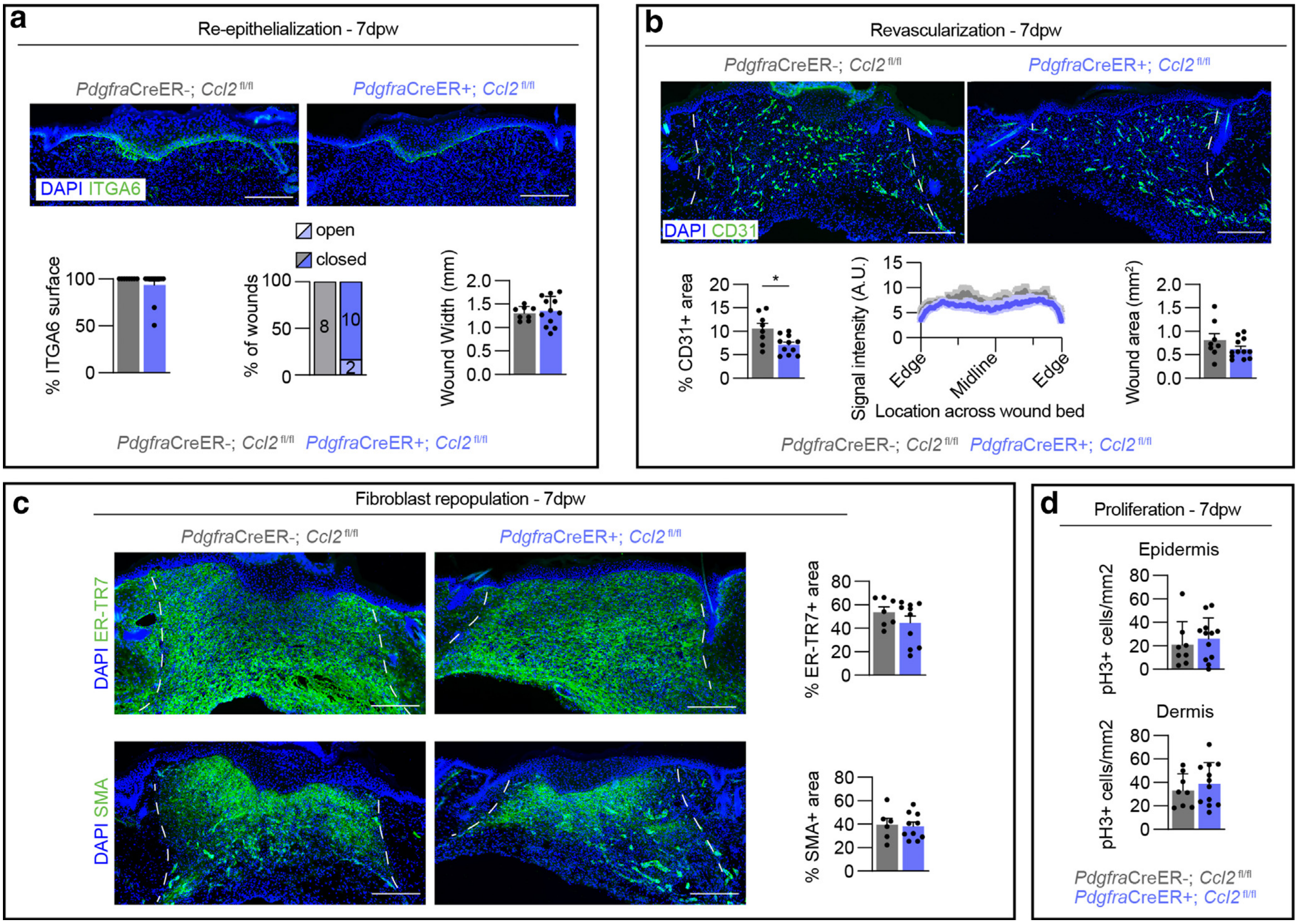

Supplementary Figure S6. Wound-healing parameters correct in FBcKO mice 7 dpw. (**a**) Images of wound beds of *Pdgfra*CreER-; *Ccl2*fl/fl (control) and *Pdgfra*CreER+; *Ccl2*fl/fl (FBcKO) mice 7 dpw immunostained for ITGA6 and DAPI. Graphs show percentage of ITGA6+ surface (left), the frequency of open or closed wounds (midline), and the wound width (right). n ≥ 8 wounds from ≥4 mice per group. (**b**) Images of wound beds of control and FBcKO mice 7 dpw immunostained for CD31 and DAPI. Graphs show the CD31+ area (left), the average distribution of CD31 signal intensity from the wound edge to the center (midline), and the total wound bed area (right). n ≥ 8 wounds from ≥4 mice per group. (**c**) Images and quantification from wound beds of control and FBcKO mice 7 dpw immunostained for ER-TR7 (top) or SMA (bottom) and DAPI. n ≥ 6 wounds from ≥3 mice per group. (**d**) Quantification of pH3+ cells in the epidermal (top) and dermal (dermal) compartments of wound beds 7 dpw in FBcKO and control mice. n = 7 wounds from 4 mice per group. Bars = 250 μm, and white lines delineate wound edges. Data points indicate individual wounds, and all data points are from male mice. Error bars indicate mean ± SEM. **P* < .05. A.U. denotes arbitrary units of fluorescence. dpw, day after wounding; FBcKO, fibroblast conditional knockout; pH3, phospho-histone H3; SMA, smooth muscle actin.

a transwell toward wound-matched CD45−CD31−SCA1+ FBs from *Pdgfra*CreER+; *Ccl2*fl/fl or *Pdgfra*CreER−; *Ccl2*fl/fl mice in the bottom of the well. FBs and monocytes/macrophages were isolated from the same wounds 1.5 dpw. n = 3 mice per group. Dotted white line corresponds to the wound edge. Triangles and circles indicate female and male mice, respectively. Error bars indicate mean ± SEM. **P* < .05 and ****P* < .001. dpw, day after wounding; DWAT, dermal white adipose tissue; FB, fibroblast; FSC-A, forward scatter area; hpw, hour after wounding; KC, keratinocyte; PC, panniculus carnosus.

Supplementary Table S1. List of Antibodies

Antibody	Source	Identifier
FITC anti-mouse CD192 (CCR2) (clone SA203G11)	BioLegend	Cat# 150608 RRID: AB_2616979
APC/Cy7 anti-mouse CD45 rat monoclonal (clone 30-F11)	BioLegend	Cat# 103116 RRID: AB_312981
FITC anti-mouse CD45 Antibody (clone 30-F11)	BioLegend	Cat# 103107 RRID: AB_312973
Alexa Fluor 700 anti-mouse CD11b rat monoclonal (clone M1/70)	BioLegend	Cat# 101222 RRID: AB_493705
eFluor 450 anti-mouse F4/80 rat monoclonal (clone BM8)	eBioscience	Cat# 48-4801-82 RRID: AB_1548747
BV785 anti-mouse Ly6G rat monoclonal (clone 1A8)	BioLegend	Cat# 127645 RRID: AB_2566317
PE/cyanine7 anti-mouse Ly-6G Antibody (clone 1A8)	BioLegend	Cat# 127618 RRID: AB_1877261
APC-Fire750 anti-mouse CD31 rat monoclonal (clone 390)	BioLegend	Cat# 102434 RRID: AB_2629683
APC/Fire 750 anti-mouse CD326 (Ep-CAM) antibody (Clone G8.8)	BioLegend	Cat# 118229 RRID: AB_2629758
APC anti-mouse CD326 (Ep-CAM) antibody (clone G8.8)	BioLegend	Cat# 118214 RRID: AB_1134102
BV650 anti-mouse Ly-6A/E (SCA1) rat monoclonal (clone D7)	BioLegend	Cat# 108143 RRID: AB_2629684
Alexa Fluor 700 anti-mouse CD29 Armenian hamster monoclonal (clone HMbeta1-1)	BioLegend	Cat# 102218 RRID: AB_493711
Anti-CD31 rat monoclonal (clone MEC13.3)	BD Bioscience	Cat# 550274 RRID: AB_393571
Anti−ER-TR7 rat monoclonal	Abcam	Cat# ab51824 RRID: AB_881651
Anti-F4/80 rat monoclonal (clone CI:A3-1)	Abcam	Cat# ab6640 RRID: AB_1140040
Anti-GFP chicken polyclonal	Abcam	Cat# 13970 RRID: AB_300798
Anti-ITGA6 rat monoclonal (clone GoH3)	R&D Systems	Cat# MAB13501 RRID: AB_2128311
Anti-Perilipin 1 goat polyclonal	Abcam	Cat# ab61682 RRID: AB_944751
Anti−phospho-Histone H3	Abcam	Cat# ab5176 RRID: AB_304763
Anti−smooth muscle actin	Abcam	Cat# ab5694 RRID: AB_2223021

Abbreviations: APC, allophycocyanin; Cat#, catalog number; PE, phycoerythrin; RRID, Research Resource Identifier.

Supplementary Table S2. qPCR Primers

Gene		Primer Sequence (5′−3′)
Actb	Forward	ATCAAGATCATTGCTCCTCCTGAG
	Reverse	CTGCTTGCTGATCCACATCTG
Ccl2	Forward	GTGCTGACCCCAAGAAGGAA
	Reverse	GTGCTGAAGACCTTAGGGCA
Ccl3	Forward	CAG CGA GTA CCA GTC CCT TT
	Reverse	GCA GTG Act GAG ACC TTC AT
Ccl5	Forward	TGCTCCAATCTTGCAGTCGT
	Reverse	GCAAGCAATGACAGGGAAGC
Ccl7	Forward	CCA TCA GAA GTG GGT CGA GG
	Reverse	TGC TTC TTG GCT CCT AGG TTG
Cxcl1	Forward	TGGCTGGGATTCACCTCAAG
	Reverse	CCGTTACTTGGGGACACCTT
Cxcl2	Forward	CACTCTCAAGGGCGGTCAAA
	Reverse	TGGTTCTTCCGTTGAGGGAC
Cxcl12	Forward	AAC ACA AGA TCC GGC AGA GG
	Reverse	ACG GCT AGG AAA GGG TCT CT
Il1a	Forward	TTGGTTAAATGACCTGCAACA
	Reverse	GAGCGCTCACGAACAGTTG
Il1b	Forward	TTGACGGACCCCAAAAGAT
	Reverse	GAAGCTGGATGCTCTCATCTG
Il6	Forward	AGCCCACCAAGAACGATAGTC
	Reverse	TTGTGAAGTAGGGAAGGCCG
Il33	Forward	CACATTGAGCATCCAAGGAA
	Reverse	AACAGATTGGTCATTGTATGTACTCAG
Tnf	Forward	AAGAGGCACTCCCCCAAAAG
	Reverse	ATCCCTTTGGGGACCGATCA
